# Transcriptomic Analysis Reveals Candidate Genes and Potential Pathways Associated with Spinal Deformity and Conjoined Twin Malformation in *Oncorhynchus mykiss* Larvae

**DOI:** 10.3390/ani16213358

**Published:** 2026-10-24

**Authors:** Yanming Sui, Yiyun Zhang, Haopeng Hu, Yuanhao Ren, Bo Qin, Na Ying, Tingting Lin, Siping Li, Hanfeng Zheng, Lei Li

**Affiliations:** 1 Key Laboratory of East China Sea Fishery Resources Exploitation, Ministry of Agriculture and Rural Affairs, East China Sea Fisheries Research Institute, Chinese Academy of Fishery Sciences, Shanghai 200090, China; suiyanming@ycit.edu.cn (Y.S.); hu15231265111@163.com (H.H.); renyh@ecsf.ac.cn (Y.R.); dhsqb1026@126.com (B.Q.); xyz6622@126.com (N.Y.); lintt@ecsf.ac.cn (T.L.); lisiping@ecsf.ac.cn (S.L.); 2 Jiangsu (Marine) Salmon and Trout Science and Technology Innovation Center, Yellow Sea Laboratory for Blue Seed Industry, Yancheng 224000, China; 3 Jiangsu Key Laboratory for Exploration and Utilization of Marine Wetland Biological Resources, Yancheng Institute of Technology, Yancheng 224051, China; zyy050106@126.com; 4 Lianyungang Ocean and Fishery Development Promotion Center, Lianyungang 222000, China

**Keywords:** *Oncorhynchus mykiss*, triploid larvae, transcriptome, spinal deformity, conjoined twin malformation

## Abstract

Rainbow trout (*Oncorhynchus mykiss*) is a globally cultured cold-water salmonid, yet triploid larvae often develop spinal ventral lordosis and conjoined twin malformations with unclear molecular mechanisms. Here, RNA-seq of 15 dph normal (N), spinal-deformed (SD) and conjoined (CT) triploid larvae yielded 54.13 Gb of valid data, identifying 4292, 3601 and 2699 DEGs for SD vs. N, CT vs. SD and CT vs. N groups, respectively. Five shared disrupted cascades (cell adhesion, neuroactive ligand-receptor interaction, phagosome, cytokine-receptor interaction, and PI3K-Akt signaling) were identified, while potential calcium/MAPK and steroid-biosynthesis-related pathways were predominantly perturbed in SD and CT larvae, respectively. Filtered PPI sub-networks displayed distinct predicted topological phenotype-associated candidate transcripts, with coagulation and complement transcripts forming core hubs in SD larvae and steroid-related transcripts showing weak connectivity in CT groups. Three discriminative transcripts with inverse expression patterns were screened. This study reveals conserved and phenotype-specific transcriptional phenotype-associated candidate transcripts of the two malformations and provides candidate phenotype-associated transcripts for subsequent functional verification of larval malformations. It should be noted that all transcriptomic data originate from whole-larval homogenates; expression signatures of conjoined-twin larvae may be confounded by altered tissue-type proportions rather than malformation-exclusive regulatory mechanisms.

## Simple Summary

Rainbow trout (*Oncorhynchus mykiss*) is an economically important farmed salmonid, but spinal ventral lordosis and conjoined twin malformations trigger heavy fry losses in triploid hatcheries. Traditional post-hatching inspection cannot identify embryonic defects early, and no parallel transcriptomic comparison of the two malformations has been reported. Here, we conducted RNA-seq on normal, spinal-deformed and conjoined twin triploid larvae, producing 54.13 Gb of valid data. Combined KEGG and filtered PPI sub-network analysis uncovered shared neuroimmune disorders, SD-specific calcium/MAPK osteogenic potential pathways and CT-unique steroid signaling. Three phenotype-associated candidate transcripts were identified. Our results reveal distinct transcriptional correlates linked to the two malformation phenotypes and provide preliminary transcriptional resources for follow-up validation studies in trout larvae. All sequencing was performed on whole-larval homogenates; transcriptional patterns observed for conjoined-twin larvae could be influenced by altered organ-tissue composition.

## 1. Introduction

Rainbow trout (*Oncorhynchus mykiss*) is a globally farmed cold-water aquaculture species with substantial economic value in fish production, recreational angling and aquatic food processing sectors [1]. Characterized by strong environmental adaptability, fast growth and superior flesh quality, it has been prioritized as a core cold-water breeding species in European, American and Asian countries [2]. Growing worldwide demand for low-fat, high-protein aquatic products has driven intensive high-value culture of *O. mykiss* throughout temperate zones and generated steady economic gains for aquaculture operators. Meanwhile, the widespread adoption of standardized recirculating and cage rearing systems has steadily lifted the annual yield of cold-water aquaculture [3]. Decades of intensive aquaculture and industrial expansion have boosted the global output of *O. mykiss*, yet larval developmental malformations have become a core bottleneck restricting the sustainable development of its breeding industry [4]. Skeletal malformations represent prevalent developmental abnormalities commonly observed in *O. mykiss* hatcheries [5]. Deformed larvae display impaired feeding and locomotion accompanied by reduced survival capacity; such abnormal individuals cannot meet commercial standards and must be manually culled, which increases labor and feeding costs and brings severe economic losses to hatcheries [6,7]. Among all larval developmental disorders observed in commercial hatcheries, spinal and conjoined malformations stand out as the most frequent and damaging phenotypes restricting fish production [8]. Embryonic development of *O. mykiss* is highly susceptible to ambient fluctuations under artificial rearing, which are associated with disrupted skeletogenesis and various skeletal lesions. Large-scale radiographic screening further confirms that vertebral malformations are ubiquitous in farmed *O. mykiss* populations globally and constitute the dominant skeletal abnormality at the larval stage [7,9]. Notably, all-female triploid *O. mykiss* have become the dominant breeding stock for intensive cold-water fish farming in China [10]. Nevertheless, molecular mechanisms underlying spinal and conjoined malformations in triploid larvae remain poorly understood. To date, no transcriptomic study has performed parallel comparisons of normal, spinal-deformed and gene regulatory networks detected as differentially expressed only within individual phenotype comparisons under the applied statistical threshold governing these two prominent malformations remain unelucidated.

Larval deformities in teleosts are correlated with complex interactions between intrinsic embryonic developmental factors and extrinsic environmental stressors, with major causative factors including environmental fluctuations, nutritional deficiencies, pathogenic infection and abnormal embryonic developmental patterning [11]. Beyond external environmental and nutritional drivers, genetic background also plays a vital role in triggering skeletal deformities in salmonids, a point that has not been sufficiently addressed in existing literature. Early inbreeding trials on rainbow trout confirmed that body curvature malformations are partially heritable, with deformed individuals appearing within full-sib mating groups, indicating the existence of heritable developmental defects [12]. Gynogenesis experiments further proved that elevated homozygosity drastically increases larval malformation rates. In doubled haploid brown trout, the proportion of deformed fry reached more than 35%, nearly three times higher than heterozygous controls, which is mainly attributed to the expression of recessive lethal alleles [13]. Similar observations across salmonid gynogenesis research further verify that high homozygosity unmasks recessive harmful variants and elevates the risk of embryonic skeletal malformations [14]. Specifically for triploid rainbow trout, spinal malformation involves more complicated combined triggers. Two unique confounding variables exist: adverse side effects caused by high hydrostatic pressure (HHP) shock during triploid induction, and distinct nutritional metabolic demands of triploid larvae compared with diploid counterparts. Thus, skeletal malformation in triploid fish results from the joint influence of genetic, operational and nutritional factors. Fjelldal et al. concluded that high water temperature acts as a primary environmental teratogen for farmed salmonids. Persistent thermal stress disrupts skeletal ontogeny and whole-body metabolic homeostasis, interferes with growth-associated signal transduction and impairs bone developmental programs, and this stress condition is closely correlated with higher incidence of permanent vertebral malformations in juveniles [15]. Poirier Stewart et al. verified that prolonged feeding on phosphorus-deficient feeds deprives juvenile all-female triploid *O. mykiss* of mineral materials essential for vertebral mineralization, which is closely associated with multiple irreversible skeletal lesions quantifiable via X-ray morphometry [16]. Madsen et al. and Ekman et al. demonstrated via field investigations and artificial-infection experiments that the cold-water pathogen *Flavobacterium psychrophilum* can invade larval vertebral and surrounding skeletal tissues, triggering sustained inflammatory infiltration; this inflammatory state is correlated with progressive bone tissue damage and irreversible vertebral lesions, while combined radiographic and pathological evidence further validated the intrinsic causal linkage between bacterial infection and progressive skeletal deformity in juvenile *O. mykiss* [17,18]. Fjelldal et al. and Boglione et al. systematically distinguished the pathogeneses of two major skeletal deformities in farmed teleosts, demonstrating that conjoined twin malformations are closely associated with arrested blastomere cleavage and aberrant embryonic body axis differentiation at the early fertilized egg stage, which are mechanistically distinct from spinal defects induced by suboptimal rearing conditions or imbalanced dietary nutrient supply [19,20]. Despite accumulated findings on extrinsic and intrinsic triggers of skeletal defects, investigations focusing on the pathogenesis of conjoined larvae remain limited. Most hatchery-oriented research merely relies on macroscopic observation and histological staining to characterize conjoined alevins across limited teleost taxa, with only sporadic field records documenting conjoined *O. mykiss* larvae; the core molecular signaling pathways closely linked to embryonic somite segmentation and twin morphogenesis remain poorly resolved through conventional histological and morphological approaches [21,22].

Given the limitations of traditional morphological observations, RNA sequencing (RNA-seq), a high-throughput transcriptomic technology, has emerged as a powerful tool to unravel the molecular basis of fish developmental defects. This platform captures the genome-wide transcriptional landscape of biological samples, enables the generation of global gene expression profiles, and aids in deciphering transcriptional regulatory networks associated with teleost embryonic development, skeletogenesis and environmental stress adaptation [22,23]. In recent years, RNA-seq has been extensively applied to decipher diverse physiological processes in *O. mykiss*. Molina et al. utilized Illumina RNA-seq to profile transcriptional changes in heat-stressed skeletal muscle of *O. mykiss*. They identified hundreds of differentially expressed genes linked to autophagy, amino acid metabolism and muscle contraction, and further clarified the core transcriptional cascades mediating muscle dysfunction associated with thermal stress in *O. mykiss* [24]. Sun et al. performed comparative hepatic transcriptome sequencing on triploid *O. mykiss* under normoxic and persistent hypoxic conditions, screened a large number of hypoxia-responsive lncRNAs as well as their target protein-coding genes, and systematically revealed key metabolic cascades that mediate hepatic adaptive responses to low dissolved oxygen [25]. Nynca et al. carried out comparative ovarian transcriptomic profiling of mature diploid and triploid female *O. mykiss*, identifying abundant differentially expressed genes linked to ovarian growth, oocyte maturation, cellular senescence and apoptotic programmes; functional enrichment analyses uncovered starkly different signaling cascades between the two ploidy groups, confirming that triploid genome architecture rewrites core gonadal developmental transcriptional programs, which are correlated with sterility in female *O. mykiss* [26]. Vehniäinen et al. constructed a cardiac transcriptomic workflow for newly hatched *O. mykiss* eleuteroembryos treated with retene, a typical AhR-agonistic polycyclic aromatic toxicant, which effectively identifies perturbed signal transduction cascades and corresponding cardiovascular developmental malformations associated with retene exposure contaminant stress [27]. Prior transcriptomic investigations of *O. mykiss* have separately addressed thermal stress in skeletal muscle, hepatic adaptation to hypoxia, ploidy-mediated gonadal regulation, and embryonic cardiotoxicity triggered by aromatic pollutants, yet few studies have unraveled the molecular mechanisms of larval skeletal malformations, creating prominent knowledge gaps in this research field. To our knowledge, no prior transcriptomic study has performed parallel multi-group comparisons of triploid *O. mykiss* larvae with three distinct phenotypes: normal, spinal-deformed and conjoined, meaning shared and phenotype-specific signaling pathways underlying the two dominant skeletal malformations have not been systematically resolved at the transcriptional level.

To fill the above research gaps, triploid *O. mykiss* larvae with three phenotypes (normal, spinal-deformed and conjoined) were sampled for pairwise transcriptional comparisons via RNA-seq. The complete analytical workflow containing quality control, DEG screening and functional enrichment was applied to mine core malformation-related genes and distinguish regulatory networks and regulatory networks detected as differentially expressed only within individual phenotype comparisons under the applied statistical threshold. The findings systematically characterize the transcriptional cascades underlying the two malformation phenotypes in triploid larvae and offer theoretical references for lowering malformation rates in hatchery production. In addition, the candidate genes and potential pathways uncovered here provide a molecular basis for future research on embryonic skeletogenesis and body axis patterning in *O. mykiss*.

## 2. Materials and Methods

### 2.1. Experimental Animals

All-female triploid *O. mykiss* were artificially propagated at the Jiangsu Ganyu Research Center, East China Sea Fisheries Research Institute, Chinese Academy of Fishery Sciences. In the case of *O. mykiss* from the cultivated broodstock, a five-minute HHP shock at 60 MPa administered 40 min after activation efficiently inhibited extrusion of the second polar body [28,29]. Before handling, the fish were anesthetized with 40 mg/L MS-222. Ripe female individuals were stripped, and fertilized eggs were collected into separate plastic containers. Broodstock used for triploid induction consisted of 6 mature female and 4 mature rainbow trout males originating from a single stable cultivated family to minimize genetic background differences. Approximately 4200 fertilized eggs were obtained by pooling stripped eggs from six mature XX females and milt from four sex-reversed XX neomales belonging to a single broodstock family, then treated with 60 MPa HHP shock to generate all-female triploid fry. All fertilized embryos, regardless of eventual phenotype, were incubated within the same standardized recirculating incubation tank system. All sequenced N, SD and CT larvae were randomly sampled from this unified egg pool, eliminating family-level or tank-derived confounding effects. All fertilized eggs originated from one unified broodstock family and were incubated in identical recirculating tanks, so malformation incidence data were not separated by family or tank. Each sequencing library was constructed from a single individual larva without tissue pooling; each conjoined twin larva was regarded as one independent biological replicate because it developed from a single fertilized egg with a shared yolk sac and identical genetic background.

The HHP treatment procedure described above was implemented using a TRC-APV electric/hydraulic apparatus (TRC Hydraulics Inc., Dieppe, Canada). Eyed eggs were incubated in recirculating freshwater tanks at a constant water temperature of 10 °C and dissolved oxygen of 8–9 mg/L under dim-light conditions following standard *O. mykiss* husbandry protocols. Moldy and non-viable eggs were discarded daily, and regular water disinfection was performed to minimize embryonic cross-contamination risks. During daily incubation management, unfertilized, moldy and developmentally arrested eggs were regularly removed. A moderate hatching loss was observed after HHP ploidy induction, which is consistent with the common developmental damage caused by high hydrostatic pressure treatment in salmonid embryos. Sufficient surviving fry covering all three target phenotypes were retained for subsequent ploidy testing and morphological classification.

Batch-level ploidy surveillance was performed by flow cytometry on randomly pre-screened fry from the whole larval batch. Separately, each of the nine larvae ultimately chosen for RNA-seq underwent individual flow-cytometry ploidy validation. Fin tissue was collected for nuclear staining using DAPI, and diploid rainbow trout fin cells were used as the reference control. Nucleus events were gated to isolate intact nuclear particles. Individuals exhibiting approximately 1.5-fold relative DNA content relative to diploid controls were classified as triploid. All larvae selected for subsequent transcriptomic analysis were individually confirmed to carry triploid genomes. At 15 days post-hatch (15 dph), all larvae were carefully observed and classified under stereomicroscopy purely based on external morphological traits into three groups: normal larvae (N), larvae showing obvious external ventral body-axis curvature (SD group, referred to as spinal-deformed for labelling purposes; no direct skeletal histological validation was performed), and ventrally fused conjoined twin larvae (CT). Individuals carrying both conjoined fusion and spinal ventral lordosis were excluded from sampling to avoid overlapping phenotype groups. Three independent biological replicates were established per group, yielding nine larval samples for subsequent transcriptomic profiling.

### 2.2. Sample Collection

Prior to sampling, all *O. mykiss* larvae were anesthetized with MS-222. Whole larval tissues were rapidly dissected, snap-frozen in liquid nitrogen immediately, and stored at −80 °C for total RNA isolation.

### 2.3. RNA Isolation, Library Construction and Illumina Sequencing

Total RNA was extracted from whole larval tissues using TRIzolTM reagent (Invitrogen, Carlsbad, CA, USA). RNA purity was measured with an ND-2000 spectrophotometer (NanoDrop, Wilmington, DE, USA), with OD260/280 values maintained between 1.8 and 2.2. RNA integrity was detected via an Agilent 5300 Bioanalyzer (Agilent Technologies, Santa Clara, CA, USA), and the RIN value of each RNA sample ranged from 7.9 to 9.2. For library construction, 1 μg total RNA was taken as the input material for each individual sample. mRNA was enriched using oligo(dT) magnetic beads to remove rRNA fragments. cDNA libraries were constructed with the SuperScript double-stranded cDNA kit (catalog number 11917-020, Invitrogen, Carlsbad, CA, USA). Purified mRNA was randomly fragmented to an insert size of ~300 bp, followed by first-strand and second-strand cDNA synthesis, end repair, single A-tailing, and adapter ligation. The cDNA fragments linked with adapters were amplified with 12 PCR cycles for library enrichment. To eliminate potential batch effects, all steps of library preparation and sequencing loading were fully randomized across N, SD and CT phenotype groups. Qualified libraries were subjected to 150 bp paired-end sequencing on the NovaSeq X Plus platform (Illumina, San Diego, CA, USA).

All nine independent larval specimens have been deposited as separate BioSamples under NCBI BioProject PRJNA1477098. Complete metadata including phenotype classification, unified brood family source, identical incubation tank conditions and non-pooled single-individual sampling information are fully annotated in the BioProject database.

### 2.4. Transcriptome Assembly and DEG Screening

Raw sequencing reads were quality-trimmed with fastp (v0.23.4) to remove adapters and low-quality reads [30]. Clean reads were mapped to the *Oncorhynchus mykiss* reference genome assembly GCF_013265735.2 (USDA_OmykA_1.1) using HISAT2 (v2.2.1) [31]. Transcript assembly and novel transcript prediction were performed using StringTie (v2.2.1) [32]. Gene raw read counts were extracted via RSEM (v1.3.3), and transcript abundance was normalized to TPM values for descriptive expression profiling only; raw count matrices rather than TPM values were imported into DESeq2 (v1.10.1) for differential statistical modeling [33]. Genes with TPM > 0 across at least one sample were defined as expressed genes. Genes with fewer than 10 total raw reads across all nine samples were pre-filtered before statistical testing to exclude low-abundance transcripts with insufficient statistical power. The design formula ~ Group was adopted, with normal larvae (N) set as the reference group for all pairwise comparisons (SD vs. N, CT vs. N). Default independent filtering was enabled in DESeq2 to improve detection power. Differential expression analyses for CT vs. N, SD vs. N and CT vs. SD were conducted using DESeq2 [34]. Genes were defined as significantly differentially expressed when |log_2_FC| ≥ 1.0 and Benjamini–Hochberg (BH)-adjusted *p*-value (*padj*) < 0.05 [34]. Regularized log (rlog) transformation of raw counts was implemented for PCA and inter-sample correlation heatmap visualization.

### 2.5. Functional Annotation and Enrichment Analysis

Homology-based functional annotation against NR, Swiss-Prot, Pfam and EggNOG databases was conducted via DIAMOND (v0.9.24) [34].

For all GO and KEGG over-representation enrichment analyses, the background gene universe comprised protein-coding genes that actually entered DESeq2 differential expression testing and possessed corresponding GO or KEGG functional annotations. Transcript and gene IDs generated from StringTie assembly were matched to orthologous entries in NR, Swiss-Prot, KEGG and GO databases via DIAMOND blastx alignment to complete uniform gene-ID orthologue mapping. Fisher’s exact test was used as the statistical test for enrichment over-representation, and the Benjamini–Hochberg procedure was applied to correct *p*-values for multiple testing. The adjusted *p*-value (*padj*) < 0.05 was set as the FDR significance threshold for all functional enrichment results. Detailed functional annotation information for all differentially expressed genes identified in this study is provided in Table S4.

Gene Ontology (GO) and Kyoto Encyclopedia of Genes and Genomes (KEGG) enrichment analyses of DEGs between different pairwise comparisons were carried out using GOATOOLS (v0.6.5) [35] and KOBAS (v2.1.1) [36], respectively. All functional annotation and enrichment results were visualized via the Majorbio Cloud platform (https://www.majorbio.com) [37].

### 2.6. Prediction of Protein–Protein Interaction Network (PPI)

The DEGs significantly enriched in key KEGG pathways from three pairwise comparisons (SD vs. N, CT vs. N, CT vs. SD) were used for PPI prediction. To further decipher interactive associations between target transcripts, full PPI networks were constructed using STRING v10.0 with default parameters for malformed *O. mykiss* larvae [38,39]. The species was designated as *O. mykiss*. The fixed prior score was set to 300, and only interactions with a combined confidence score ≥ 0.5 were retained. Experimental and database-supported interactions were reserved, while text-mining predicted links were discarded to reduce false positives. All input DEGs were converted to STRING-compatible protein IDs, and the numbers of mapped and unmapped transcripts for each group were summarized in supplementary tables. Full networks containing all qualified high-confidence interactions were generated without manual edge filtering. Afterwards, each full network was filtered to retain only the top 300 highest-confidence interaction edges. These filtered subnetworks (top 300 edges) were used for all topological-parameter calculations and hub-gene identification in Cytoscape v10.0.

### 2.7. Screening of Phenotype-Associated Candidate Transcripts

Transcripts were screened using multi-layer filtering criteria. Transcripts with |log_2_FC| ≥ 1 and *padj* < 0.05 in at least one pairwise comparison (SD vs. N, CT vs. N, CT vs. SD) and complete KO and KEGG pathway annotations were retained initially. Only genes assigned to malformation-relevant KEGG pathways were further retained. Subsequent filtering reserved transcripts with divergent expression profiles between SD and CT groups. A large number of transcripts passed all screening thresholds. Thirteen representative transcripts with diversified functional annotations and non-redundant expression patterns were selected from qualified candidates. Three candidate transcripts were further identified for their inverse expression trends between the two malformed groups.

### 2.8. Statistical Analysis

PCA plots, Venn diagrams, gene clustering heatmaps and enrichment bubble charts were generated using R software (v4.2.1) and the Majorbio Cloud platform (https://www.majorbio.com). All data are presented as mean ± standard deviation (SD) of three biological replicates, and differences at *p* < 0.05 were considered statistically significant.

## 3. Results

### 3.1. Observation of External Morphological Phenotypes of Larvae

**Figure 1:**
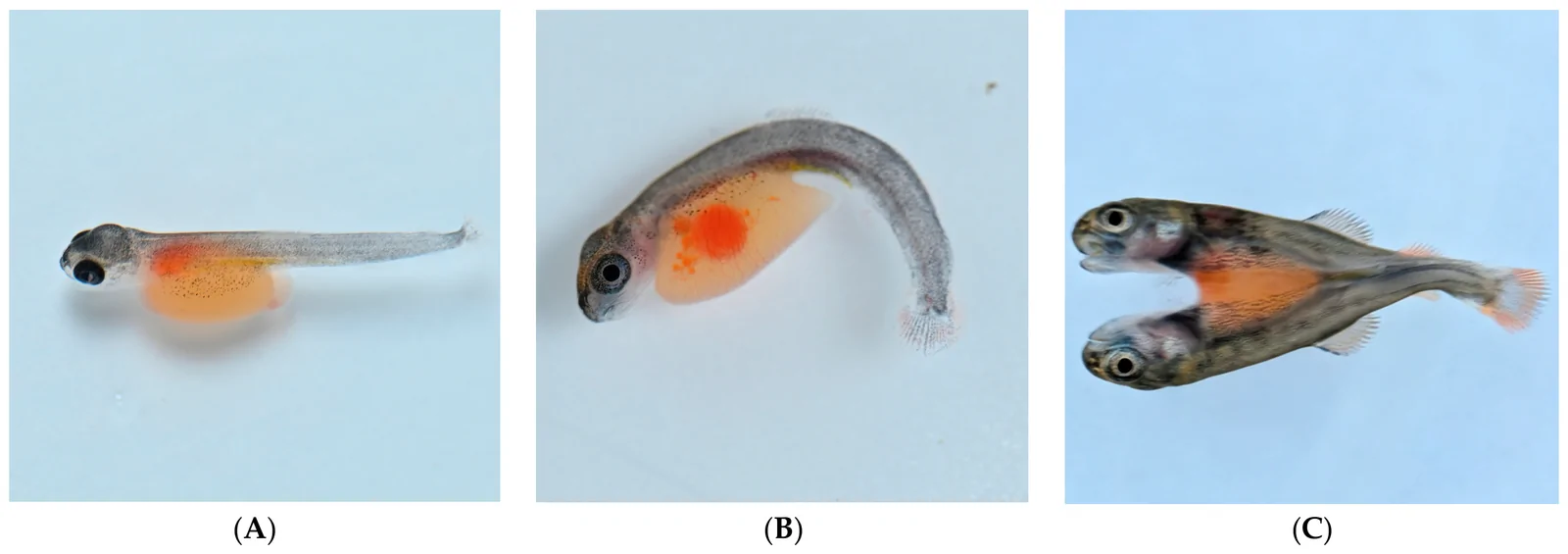
Stereomicrographs of three types of 15 dph triploid *O. mykiss*. (**A**) N, normal larva with straight and symmetric vertebral trunk. (**B**) SD, larva with obvious ventral external ventral body-axis curvature. (**C**) CT, ventrally fused conjoined twin larvae sharing a single yolk sac.

To characterize larval phenotypic differences, 15 dph yolk-sac larvae were screened under a stereomicroscope and classified into three groups abbreviated N, SD and CT (Figure 1). N larvae possessed slender, bilaterally symmetric trunks with intact vertebral columns (Figure 1A). Their heads were fully developed with paired uniform eyes, accompanied by well-proportioned yolk sacs and complete dorsal and caudal fins, and the larvae exhibited stable, coordinated swimming behavior. SD larvae exhibited obvious ventral lordosis along the mid-trunk, disrupting bilateral body symmetry (Figure 1B). Although cranial development remained normal, spinal malformation impaired balance and swimming performance. CT larvae were ventrally fused and shared a single yolk sac, with two independent heads, pectoral fins and caudal fins (Figure 1C). Synchronous spinal curvature was detected on fused trunk segments, along with reduced locomotion and feeding ability. A small number of CT larvae exhibited faint curvature on fused trunk segments. Individuals showing both obvious conjoined fusion and distinct severe external ventral body-axis curvature matching SD group criteria were strictly defined as mixed-phenotype and excluded from transcriptome sequencing to guarantee clear group separation. Minor faint trunk curvature that did not fulfil SD group severity thresholds was tolerated within the CT group.

### 3.2. Quality Control of Transcriptome Sequencing Data

Transcriptome sequencing was conducted using nine larval replicates to profile transcriptional differences across N, SD and CT groups. After quality filtering, a total of 54.13 Gb clean data was generated. The Q30 value of each library exceeded 95.3% (Table S1). Clean reads were mapped to the *O. mykiss* reference genome, with mapping rates ranging from 94.11% to 94.69% (Table S2).

### 3.3. Sample Correlation and Principal Component Analysis

**Figure 2:**
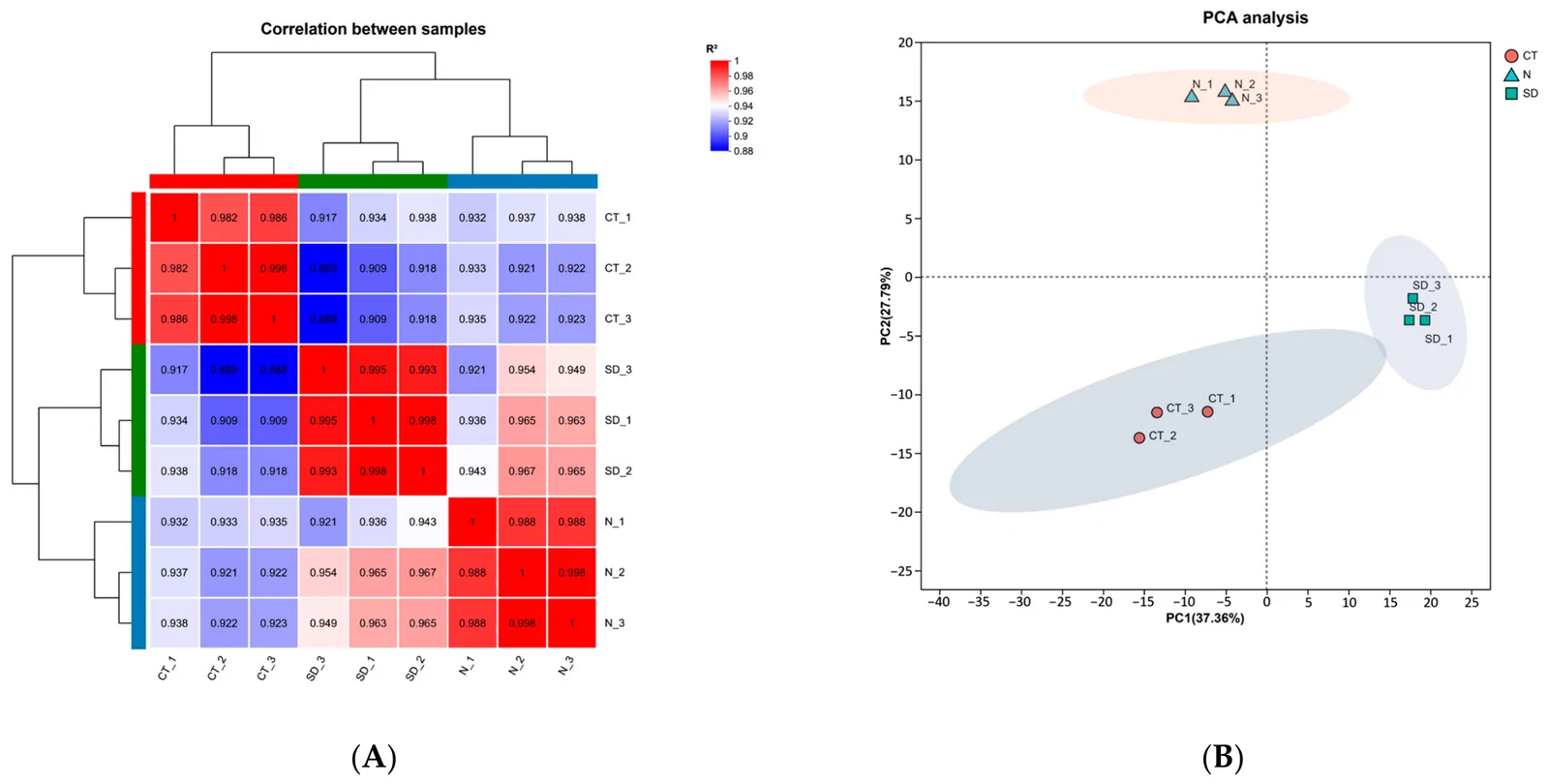
Transcriptome-wide sample correlation and PCA analysis. (**A**) Heat map illustrating pairwise gene expression correlation coefficients across all nine biological replicates. (**B**) PCA scatter plot (triangle = N larvae, square = SD larvae, circle = CT larvae). PC1 explains 37.36% and PC2 explains 27.79% of total transcriptional variance.

Gene expression correlations and principal component (PCA) analysis based on rlog-transformed count matrices were computed to assess inter-sample reproducibility and transcriptional divergence. TPM values were only used for descriptive expression profiling. The correlation heatmap showed that correlation coefficients of biological replicates within each group were all higher than 0.88 (Figure 2A). PCA clearly separated the N, SD and CT groups without overlapping clusters, demonstrating distinct transcriptional divergence across the three larval phenotypes (Figure 2B).

### 3.4. Identification of Differentially Expressed Genes

**Figure 3:**
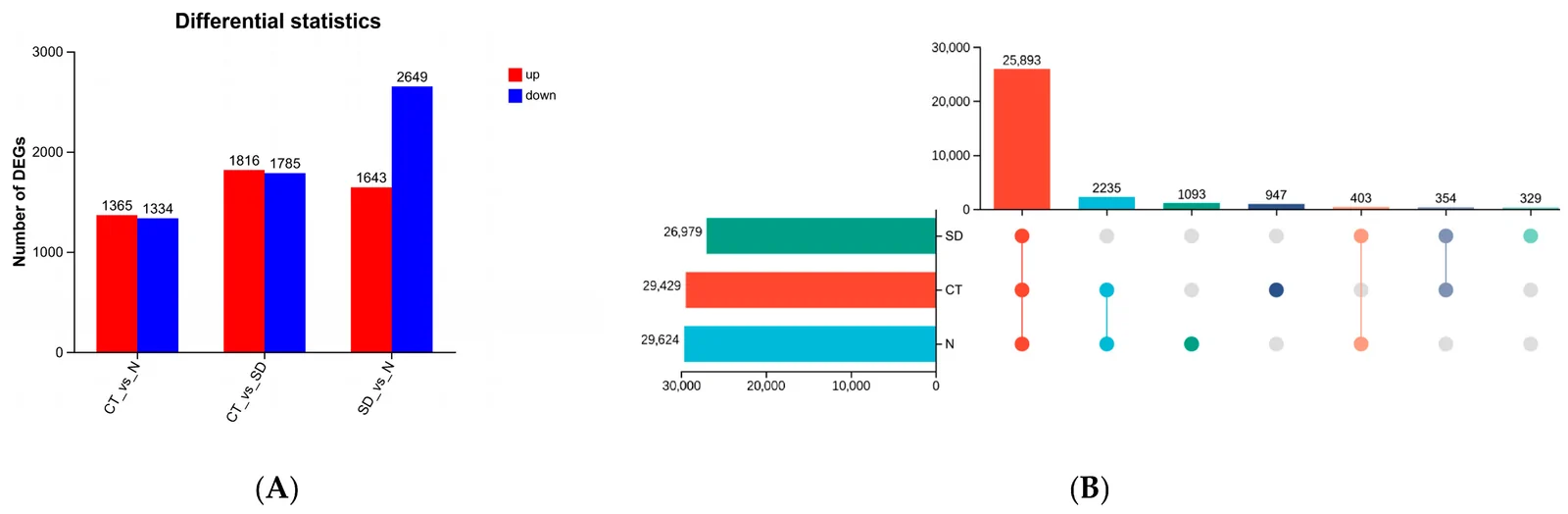
Summary of DEGs across three pairwise comparisons. (**A**) Number of DEGs identified between N, SD and CT groups. Red color indicates up-regulated genes and blue color indicates down-regulated genes in each comparison, respectively. (**B**) UpSet plot illustrating overlaps of differentially expressed genes derived from three pairwise transcriptome comparisons. Vertical bars indicate the number of DEGs for each intersection category.

To screen genes with altered expression between phenotypes, differential expression analysis was carried out. A total of 4292 DEGs were identified in SD vs. N, 2699 DEGs in CT vs. N, and 3601 DEGs in CT vs. SD (Figure 3, Table S3). The SD vs. N comparison yielded the greatest number of DEGs, including 1643 upregulated and 2649 downregulated transcripts. CT vs. N harbored 1365 upregulated and 1334 downregulated genes, whereas CT vs. SD yielded 1816 upregulated and 1785 downregulated DEGs. An UpSet plot was constructed to display shared and unique DEGs across all three pairwise comparisons. There were 403 DEGs detected as differentially expressed only in the SD vs. N comparison under the applied statistical threshold, 2235 DEGs detected as differentially expressed only in the CT vs. N comparison under the applied statistical threshold, and 354 DEGs detected as differentially expressed only in the CT vs. SD comparison under the applied statistical threshold (Figure 3B).

### 3.5. GO Enrichment Analysis of DEGs

**Figure 4:**
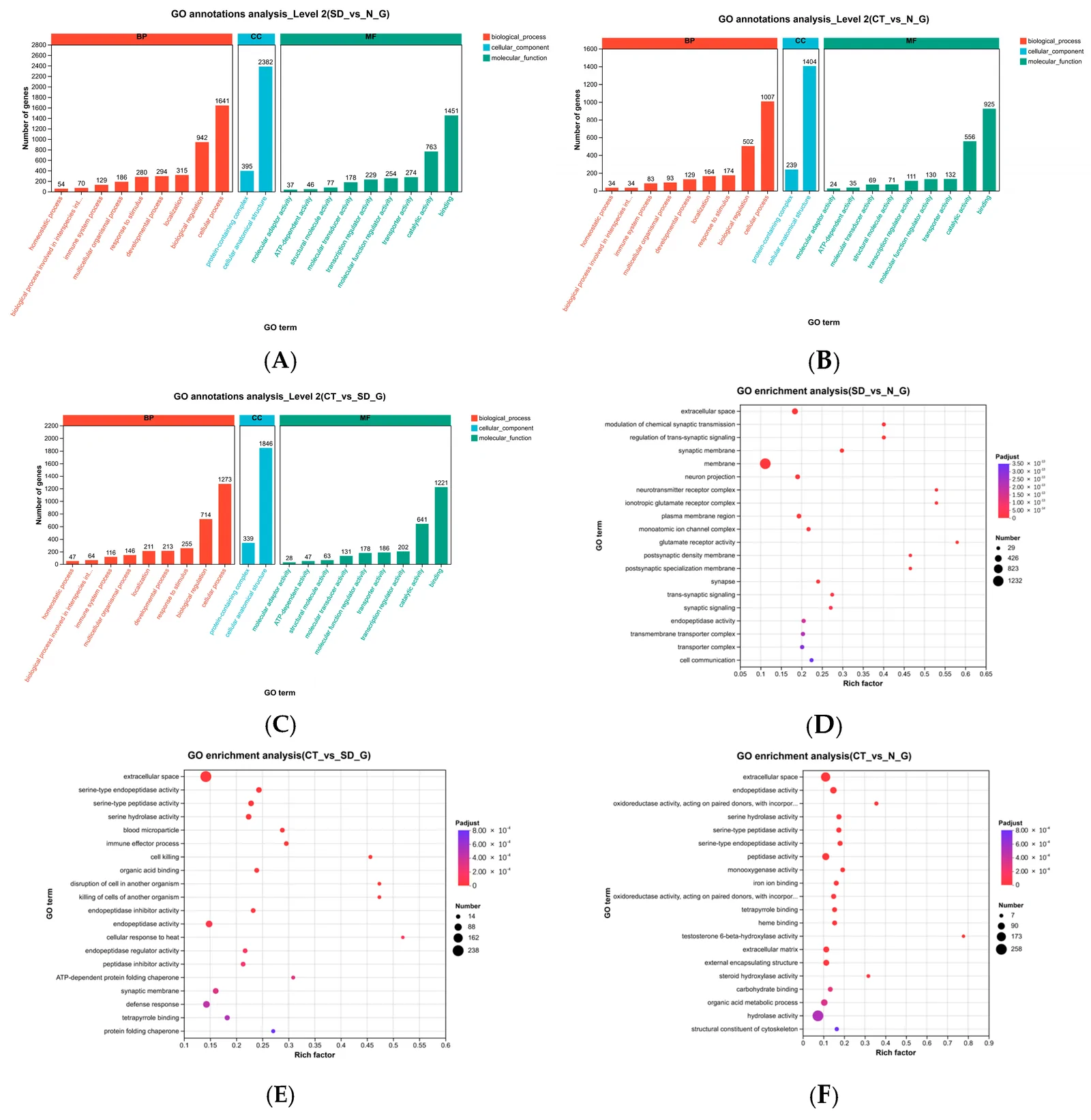
GO enrichment analysis of DEGs. (**A**) Global GO functional classification for the SD vs. N comparison. (**B**) Global GO functional classification for the CT vs. N comparison. (**C**) Global GO functional classification for the CT vs. SD comparison. (**D**) Top 20 significantly enriched GO terms in the SD vs. N comparison. (**E**) Top 20 significantly enriched GO terms in the CT vs. N comparison. (**F**) Top 20 significantly enriched GO terms in the CT vs. SD comparison.

The functional characterization of the DEGs was performed through Gene Ontology (GO) enrichment analysis across biological process (BP), cellular component (CC), and molecular function. To fully interpret the enrichment results, analyses were implemented at two levels: overall functional classification (Figure 4A–C) and screening of the top 20 significantly enriched GO terms (Figure 4D–F, Tables S5–S7). Cellular anatomical structure, cellular processes and molecular binding constitute the dominant functional categories enriched with DEGs in all three comparisons. For SD vs. N, the top significantly enriched GO terms were extracellular space, synaptic membrane, modulation of chemical synaptic transmission and regulation of trans-synaptic signaling (Figure 4D, Table S5). or CT vs. N, prominently enriched terms included extracellular space, endopeptidase activity, and oxidoreductase activity acting on paired donors (Figure 4E, Table S6). For CT vs. SD, DEGs were markedly enriched in extracellular space, serine-type endopeptidase activity, immune effector process and cell killing (Figure 4F, Table S7).

### 3.6. KEGG Pathway Enrichment Analysis

**Figure 5:**
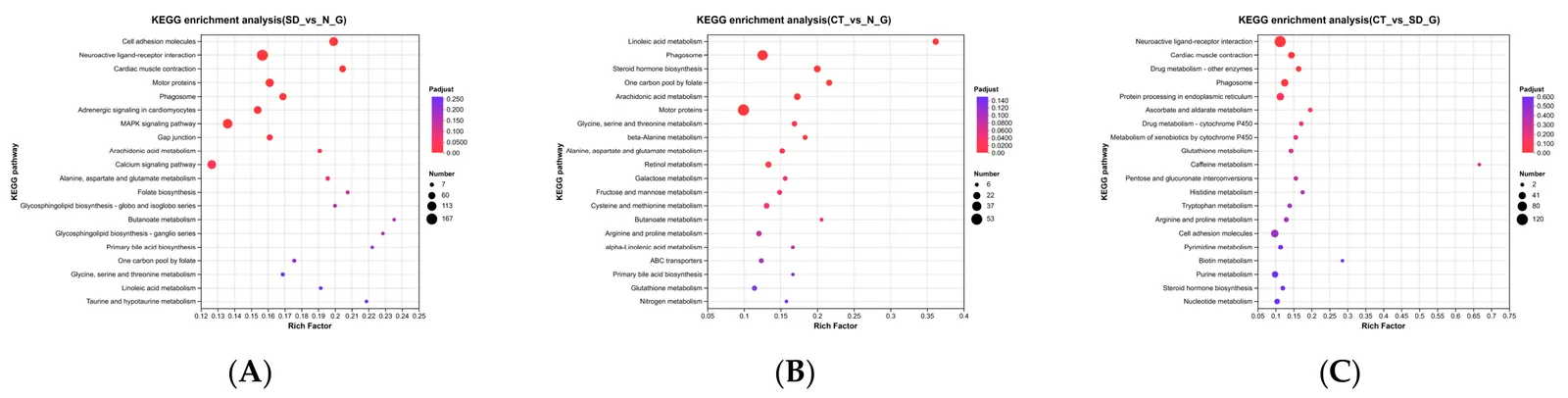
Ranking of the top 20 KEGG pathways ordered by enrichment significance for each pairwise DEG comparison. (**A**) Top-ranked KEGG pathways of SD vs. N DEGs; (**B**) Top-ranked KEGG pathways of CT vs. N DEGs; (**C**) Top-ranked KEGG pathways of CT vs. SD DEGs. Bubble size corresponds to the number of DEGs enriched in each pathway. Note that this figure displays pathways ranked by significance rank only; not all entries meet the *padj* < 0.05 significance threshold.

To further dissect the biological functions of DEGs at the pathway level, we performed KEGG enrichment analysis and generated bubble plots to visualize the top 20 significantly enriched pathways for each pairwise comparison (Figure 5, Tables S8–S10). Note that not all plotted pathways satisfy the *padj* < 0.05 significance threshold. For SD vs. N, the most significantly enriched pathways included cell adhesion molecules, neuroactive ligand-receptor interaction, phagosome, MAPK signaling pathway and calcium signaling pathway (Figure 5A, Table S8). For CT vs. N, DEGs were highly enriched in linoleic acid metabolism, arachidonic acid metabolism, steroid hormone biosynthesis, phagosome and motor proteins (Figure 5B, Table S9). For CT vs. SD, enriched pathways covered neuroactive ligand-receptor interaction, cardiac muscle contraction, phagosome, protein processing in endoplasmic reticulum and metabolism of xenobiotics by cytochrome P450 (Figure 5C, Table S10).

To rigorously identify shared perturbed pathways and pathways detected as significantly enriched only within individual pairwise comparisons under the applied statistical threshold, we performed cross-comparison of all KEGG terms with *padj* < 0.05 across the three pairwise datasets, rather than only referencing the top 20 ranked pathways shown in Figure 5. Pathways showing significant enrichment (*padj* < 0.05) in both SD vs. N and CT vs. N comparisons were defined as shared perturbed cascades. Significance in the CT vs. SD contrast is not required for shared-response classification, as this contrast highlights transcriptional differences between the two malformation phenotypes. Pathways with significant enrichment were detected as significantly enriched only in the SD vs. N comparison under the applied statistical threshold; pathways with significant enrichment were detected as significantly enriched only in CT-related comparisons under the applied statistical threshold. Separate enrichment analyses of exclusively upregulated and exclusively downregulated DEG subsets further distinguished pathway activation or suppression, eliminating biased interpretations derived from over-representation tests using mixed-direction DEGs.

### 3.7. Protein–Protein Interaction Analyses (PPI)

**Figure 6:**
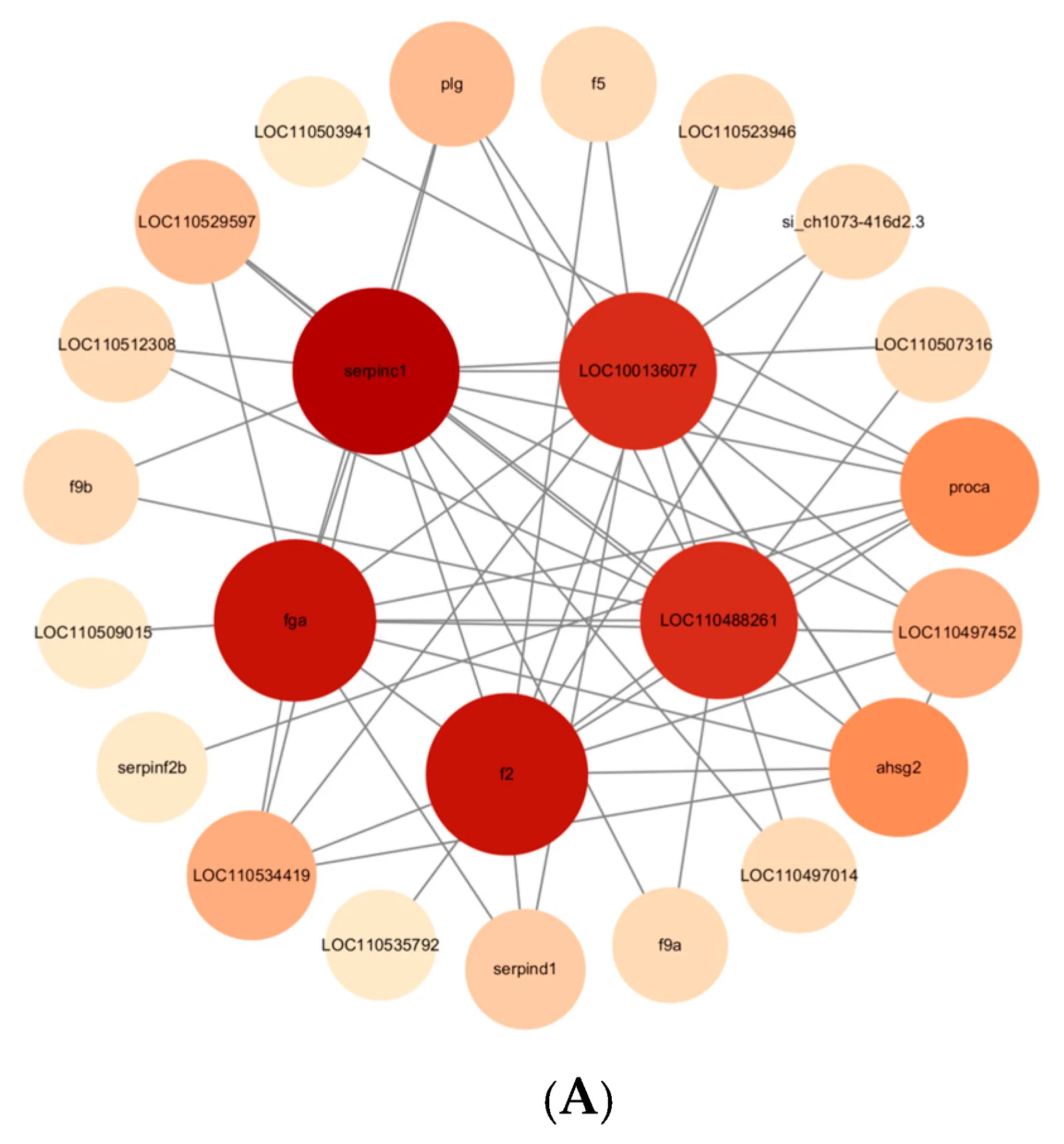

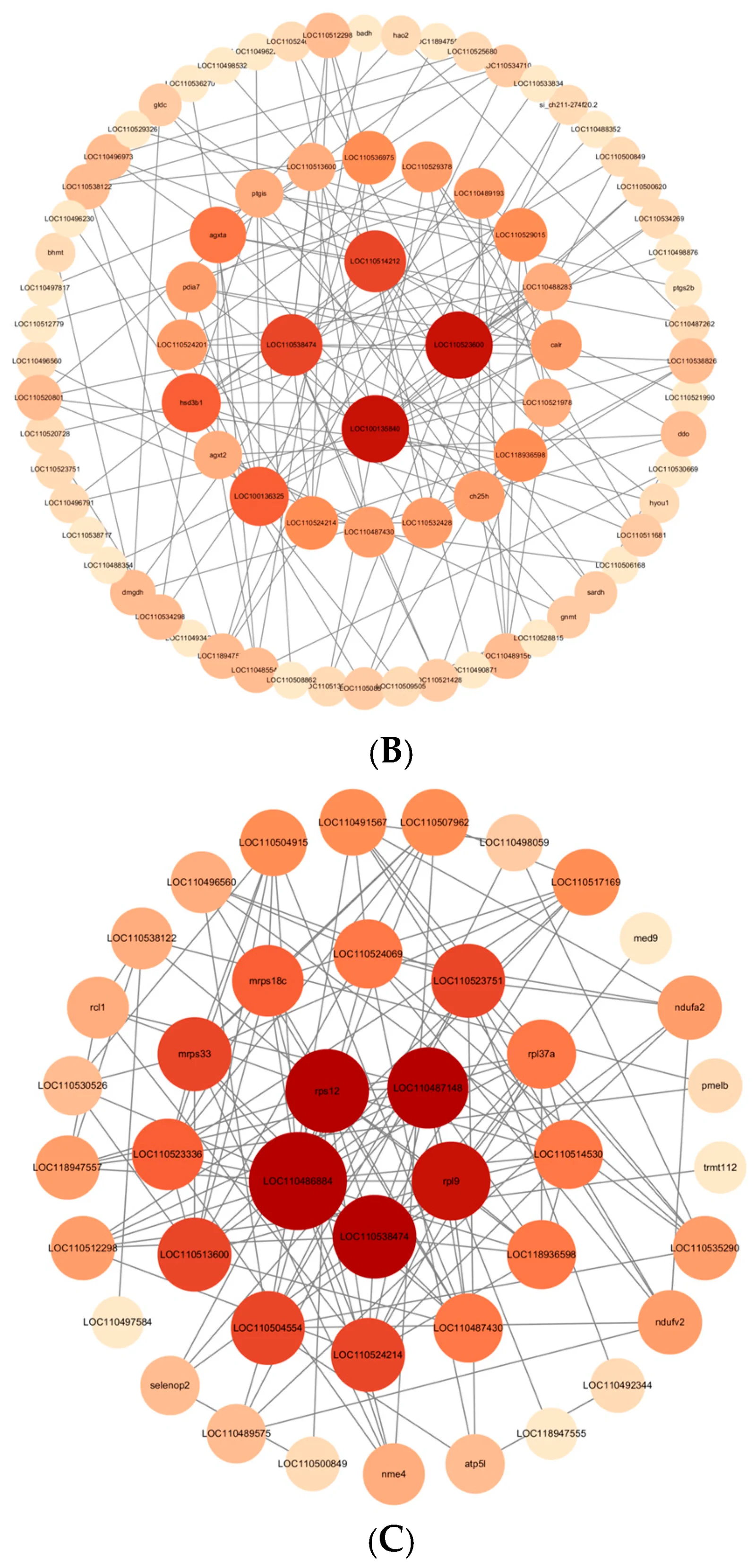
Core modules extracted from top-300-edge PPI sub-networks of three pairwise comparison groups in triploid *O. mykiss* larvae. (**A**) PPI sub-network of SD vs. N group. (**B**) PPI sub-network of CT vs. N group. (**C**) PPI sub-network of CT vs. SD group.

To further dissect core interactive gene modules underlying spinal and conjoined malformations, full PPI networks were built for SD vs. N, CT vs. N and CT vs. SD groups based on KEGG-enriched DEGs. Each full network was subsequently filtered to retain only the top 300 highest-confidence interaction edges. These filtered subnetworks were used for all topological quantification and hub-gene screening (Figure 6).

The SD vs. N full network contained 257 nodes and 300 edges, with an average node degree of 2.335, an average shortest path length of 1.809, an average clustering coefficient of 0.368, and a transitivity of 0.361. Multiple high-centrality core transcripts constituted a tightly linked immune-coagulation functional module, among which *serpinc1* (degree = 13) exhibited the highest connectivity, followed by coagulation-associated *f2* (degree = 12), fibrinogen-related *fga* (degree = 12), and complement factor genes *c5*, *c8a*, *c9*. The T-cell-marker transcript *cd3e* was also detected in the full network but exhibited low node connectivity (degree = 3). To simplify visualization, this gene was omitted from the condensed PPI graph (Figure 6A).

The CT vs. N full network comprised 268 nodes and 300 edges, with an average node degree of 2.239, an average shortest path length of 3.409, an average clustering coefficient of 0.445, and a transitivity of 0.350. Its core interactive cluster centered on two high-degree unannotated transcripts *LOC100135840* and *LOC110523600* (both degree = 12). Meanwhile, multiple metabolism- and immune-related genes were distributed around this hub cluster, including steroid metabolic genes *ch25h*, *hsd3b1*, protein folding factor *pdia7*, and amino acid metabolic transcripts *gldc*, *dmgdh* (Figure 6B).

The CT vs. SD full network included 207 nodes and 300 edges, with an average node degree of 2.899, an average shortest path length of 2.616, an average clustering coefficient of 0.568, and a transitivity of 0.532. Ribosomal protein transcripts formed the dominant dense core module of this comparison group; *rps12* possessed the maximum node (degree = 14), and its direct interaction partner *rpl9* also showed high connectivity (degree = 12). Besides ribosome hubs, mitochondrial ribosomal genes *mrps18c*, *mrps33* and complement component *c9*, *c8a*, *c5* also formed secondary small interactive clusters in the full network (Figure 6C).

### 3.8. Expression Patterns of Signature DEGs Related to Larval Malformation

**Figure 7:**
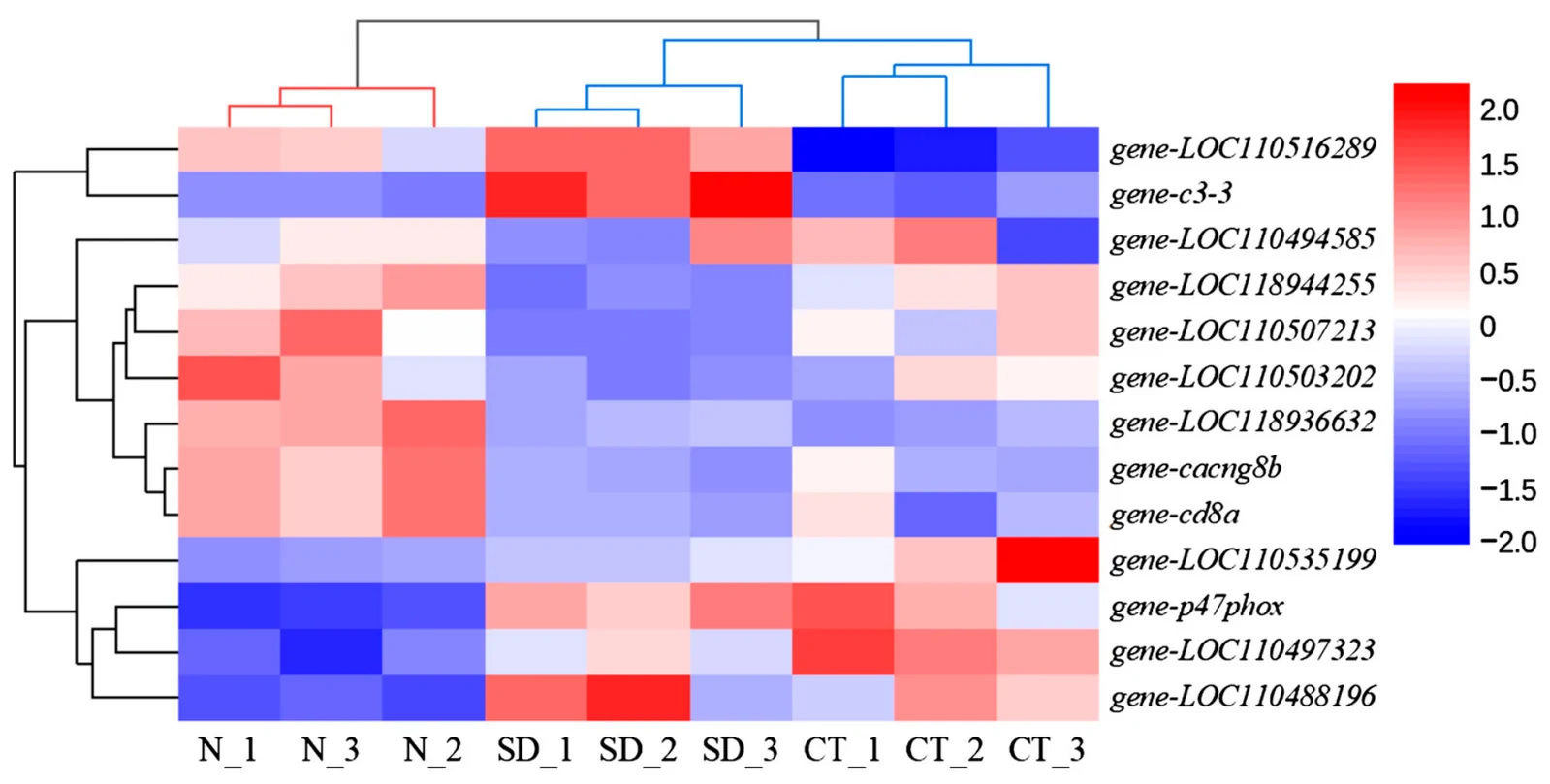
Heat map illustrating the relative expression of phenotype-associated candidate transcripts and malformation-related DEGs. Rows represent candidate functional genes, and columns correspond to larval biological replicates: N1–N3 (normal larvae), SD1–SD3 (spinal-deformed larvae), CT1–CT3 (conjoined-twin larvae). Gene expression values were normalized via row-wise Z-score transformation. Red indicates high relative expression, and blue indicates low relative expression.

To further characterize the representative DEGs enriched in the top KEGG pathways across three pairwise comparisons (SD vs. N, CT vs. N, CT vs. SD), we extracted phenotype-associated candidate transcripts from cell adhesion, neuroactive ligand-receptor interaction, calcium signaling, steroid biosynthesis, lipid metabolism and phagosome pathways for integrated analysis, and their normalized expression profiles were visualized in a single heatmap (Figure 7 and Figure S1, Table S10). The screened phenotype-associated candidate genes included *col9a* (LOC110488196), *cd8a*, *grm5* (LOC110507213), *cnr1* (LOC118944255), *cacng8b*, *cyp21a* (LOC110535199), *cyp3a* (LOC118936632), *cyp2u1* (LOC110497323), *c3-3*, *ncf1* (p47phox), *anpep* (CD13, LOC110516289), *slitrk3* (LOC110503202) and *itpr1* (LOC110494585).

For the comparison between SD and normal N larvae, the cell adhesion gene *col9a* showed a trend toward up-regulation, while the immune adhesion marker *cd8a* exhibited a trend toward down-regulation. Two synaptic receptor genes, *grm5* and *cnr1*, were suppressed within the neuroactive ligand-receptor interaction pathway, and calcium channel genes exhibited variable expression levels in lordotic SD larvae. In contrast, when comparing CT against normal larvae, steroid biosynthetic genes displayed opposite expression trends: *cyp21a* was highly induced whereas *cyp3a* was significantly repressed, with divergent steroid gene expression detected in conjoined larvae; the lipid metabolism gene *cyp2u1* was also markedly elevated in CT larvae. Regarding phagosome-related immune factors, *c3-3* and *anpep* were downregulated while *ncf1* was upregulated. Further comparison between CT and SD revealed that the synaptic receptor *grm5* maintained higher expression in CT individuals; the two immune genes *c3-3* and *anpep* showed lower transcript abundance in CT, whereas the cell adhesion gene *slitrk3* and calcium receptor *itpr1* were upregulated.

## 4. Discussion

### 4.1. Global Transcriptional Remodeling and Conserved Molecular Signatures of Developmental Defects

Triploid *O. mykiss* larvae possess inherent developmental fragility during embryogenesis, and skeletal malformations will severely retard their growth and sharply raise mortality. To address this research gap, we conducted parallel RNA-seq profiling across normal, SD and CT triploid larvae, since prior work has not systematically defined shared and phenotype-specific transcriptional phenotype-associated candidate transcripts of the two malformations [8,40]. Global transcriptome-wide profiling uncovered clear transcriptional divergence between malformed and normal larvae. Under our applied statistical thresholds, a larger total number of DEGs was detected for the SD vs. N contrast compared with the CT vs. N contrast. Note that raw DEG counts are affected by multiple technical and statistical factors and cannot be directly interpreted as reflecting the biological magnitude of molecular perturbation. Consistent with our transcriptomic observations, Amoroso et al. documented broad immune- and skeletal-related transcriptional dysregulation in triploid *Salmo salar* with jaw malformations; this evidence further verifies that genome-wide transcriptional perturbations are widespread in triploid salmonids [41].

KEGG functional annotation of all DEGs categorized perturbed signaling cascades into two primary classes: shared potential pathways disrupted in both malformation groups, and regulatory axes detected as differentially expressed only within individual phenotype comparisons under the applied statistical threshold for SD or CT larvae. Five core neuroimmune- and cell adhesion-related pathways exhibited significant transcriptional correlation with both malformation phenotypes in our enrichment analyses [42]. In contrast, calcium and MAPK pathway transcripts showed unique transcriptional correlations to spinal-deformed larvae, while steroid biosynthesis-related genes displayed distinct expression shifts associated with conjoined twin malformation [43,44]. The divergent transcriptional phenotype-associated candidate transcripts observed between the two malformation subtypes imply potential distinct embryonic developmental correlates. However, these transcriptome-wide associations are limited by the use of whole-larval homogenates, which cannot resolve tissue-specific transcriptional signals. Most prior salmonid omics research only characterized a single skeletal deformity, with few systematic summaries of such divergent transcriptional profiles available [45].

Accumulated mutant research on zebrafish models of spinal curvature has demonstrated that mechanosensitive Piezo ion channels (piezo1, piezo2) are critical regulators of vertebral patterning and bone mineralization; disrupted Piezo channel function leads to juvenile-onset axial scoliosis and asymmetric vertebral growth, consistent with the skeletal dysregulation observed in spinal-deformed rainbow trout larvae [46,47]. Conserved mechanical sensing signaling between teleost species may partially explain the phenotype-specific enrichment of calcium and MAPK cascades detected in SD triploid larvae.

### 4.2. Correlative Immune-Related Transcriptional Signals Inferred from Cross-Species Literature and In-Silico PPI Evidence

Genes with core immune regulatory functions were repeatedly recovered from shared modules of the three filtered PPI sub-networks. This finding indicates immune-related transcripts display coordinated expression shifts linked to larval developmental defects, without evidence of sustained immune dysfunction. The T cell marker transcript cd3e was identified in the full PPI networks of the SD vs. N and CT vs. N comparisons but exhibited low node connectivity, demonstrating that it is not a central hub within these predicted interaction modules.

Isles et al. conducted developmental experiments on *Danio rerio* and uncovered that *cxcl12a* exerts dual biological roles to coordinate embryonic tissue patterning and directional immune cell migration [48]. Nevertheless, no high-confidence interaction pairs associated with *cxcl12a* were detected in our filtered PPI datasets. Current topological data cannot fully resolve its upstream and downstream signaling cascades; therefore, deeper interactome profiling is required in subsequent research. Transcriptional changes in core immune transcripts observed in malformed larvae correlate with altered immune-related pathway profiles, which may point to developmental immune imbalance associated with larval malformation phenotypes. Comparative proteomic profiling carried out by Fan et al. on conjoined *Gambusia affinis* further validated that this immune dysregulation pattern is highly conserved across teleost evolution [49]. We speculate that the coordinated transcriptional shifts in immune pathways may represent a correlative transcriptional response linked to structural developmental anomalies in malformed larvae, though tissue-specific and functional evidence is lacking to confirm this feedback hypothesis.

In addition to T cell- and chemokine-related transcripts, multiple members of the CXC/CC chemokine superfamily and *socs3* displayed prominent transcriptional alterations in malformed larvae. CXC and CC chemokines function as core effector mediators of innate immunity across teleosts [50]. Related transcriptomic analyses of *Ctenopharyngodon idella* and *Oplegnathus fasciatus* verified that *socs3* mediates negative feedback regulation of inflammatory cytokine signaling under pathogen challenge [51,52]. Among these immune mediators, the regulatory linkage between *cxcr4* and the stress signaling axis has been verified in *Cyprinus carpio*, which may partly account for the distinct transcriptional alterations of *cxcr4* observed in malformed larvae of the present study [53]. Combined with our multi-omic datasets, this study further suggests that embryonic malformation correlates with strong transcriptional perturbation of *cxcr4* in salmonid larvae, a phenomenon rarely documented in previous research on triploid *O. mykiss*. From a functional perspective, the *cxcr4* signaling axis modulates two key biological processes: immune cell chemotaxis and embryonic neuronal migration accompanied by tissue remodeling [54,55]. Collectively, shared immune dysfunction in both malformation subtypes stems from coordinated transcriptional alterations of various chemokines and T cell marker genes. Additional functional validation experiments are required to clarify the precise regulatory roles of these core immune hub genes during teleost embryonic lesions.

### 4.3. Phenotype-Specific Regulatory Cascades and Distinct PPI Topological Modules

All topological metrics and hub-gene identification were calculated from subnetworks constrained to top-300 highest-confidence edges. This artificial edge-number restriction may introduce structural network bias; differences in topological parameters among groups should be interpreted with considerable caution and cannot be directly taken as solid biological evidence. Calcium and MAPK signaling cascades are evolutionarily conserved core modules regulating chondrocyte maturation and vertebral mineralization in teleosts. Findings from related skeletal malformation research confirm that both cascades become perturbed under developmental bone lesions [43]. Consistent with pathological traits of SD triploid *O. mykiss* larvae, PPI topological analysis showed immune-coagulation gene clusters form core interaction hubs detected only within SD-relevant comparisons under the applied statistical threshold. Co-expressed inflammatory gene modules correlated with spinal deformity-related nodes in the PPI network, a correlative transcriptional pattern consistent with the transcriptomic findings described by Li et al. [43].

In contrast, steroid biosynthesis pathway genes exhibited distinct expression correlations detected as differentially expressed only in CT-related comparisons under the applied statistical threshold in conjoined twin larvae, and this pathway has been reported to participate in embryonic germ layer and body axis patterning across teleost species in prior work [56]. Morphological observations of conjoined salmonid larvae confirmed arrested early blastomere cleavage as the primary trigger of embryonic fusion [57]. Comprehensive reviews addressing vertesteroid biofunctions further illustrate that coordinated interactions between steroid synthases and tissue remodeling factors dominate developmental programs specific to conjoined embryos [56]. Divergent transcriptional patterns among ribosomal and steroid-related transcripts constitute one set of distinguishable molecular correlations observed between the two malformation groups at the transcript level. Within these edge-restricted subnetworks, distinct sets of hub genes were observed. Immune- and coagulation-related transcripts occupied central positions in the SD-relevant subnetwork; steroid-metabolism-associated transcripts were prominent among hub nodes in the CT vs. N subnetwork, whereas ribosomal-related transcripts constituted major hub nodes in the CT vs. SD subnetwork. These are observational patterns within edge-filtered subnetworks and should not be generalized as definitive biological signatures for malformation subtypes.

Integrated KEGG enrichment and hub gene screening from filtered PPI sub-networks identified three phenotype-discriminative transcripts. *col9a* encodes a core extracellular matrix protein that controls notochord mineralization, and its differential expression serves as a specific molecular phenotype-associated candidate transcript of vertebral developmental lesions [44]. *cd8a* and *grm5* display opposing expression patterns between SD and CT groups. Such divergent transcriptional profiles are consistent with transcriptomic data of cold-induced skeletal malformation in spotted sea bass reported by Pan et al. [42].

### 4.4. Biomarkers, Production Value, Limitations and Future Perspectives

This study represents an exploratory transcriptomic investigation, with only three biological replicates for each phenotype group. Owing to the rarity of these spontaneously occurring malformed larvae, additional matched independent samples could not be acquired. A small sample size may reduce statistical power and robustness when identifying phenotype-associated transcripts; accordingly, all transcriptomic associations observed in this work should be interpreted with caution. This study identifies several phenotype-associated candidate transcripts. Since sampling was conducted after malformation phenotypes were already visible, we did not perform independent cohort testing or diagnostic performance assessment. These transcripts are merely preliminary candidates derived from transcriptomic data, and multi-group experimental validation will be required in follow-up research. Another important confounding factor should be noted: RNA-seq was performed using whole-larval homogenates. For CT conjoined-twin larvae, alterations in relative organ and tissue proportions compared to normal single larvae may contribute to observed differential expression signals. Therefore, transcriptional differences identified in CT-related comparisons cannot be exclusively attributed to molecular mechanisms driving conjoined-twin malformation and may be partly caused by shifts in tissue composition. Caution should be exercised when inferring causal developmental mechanisms underlying conjoined-twin phenotypes based solely on whole-larva transcriptomic data. Calcium and neuroactive ligand-receptor pathways show conserved transcriptional correlations with developmental anomalies across teleost larval models in published omics research, as verified by transcriptomic data from hypoxia-exposed *Hypophthalmichthys molitrix* and heat-challenged *C. idella* [58,59].

The phenotype-associated hub genes identified in this study may provide preliminary molecular clues for follow-up embryonic screening research in triploid trout hatcheries. High malformation ratios cause substantial economic losses in triploid aquaculture, generating an urgent demand for embryonic molecular phenotype-associated candidate transcripts; cross-species multi-omic analyses confirm these DEGs can be developed into rapid molecular detection assays [8,40].

The present study has inherent experimental limitations. Firstly, total RNA was extracted from whole-larval homogenates instead of lesion-specific tissues. Consequently, tissue-specific transcriptional phenotype-associated candidate transcripts at damaged regions cannot be detected, and tissue-targeted transcriptome sequencing can significantly enhance the analytical resolution of skeletal injury-related signaling cascades. Secondly, all gene associations obtained from RNA-seq and PPI prediction merely show correlative relationships. qRT-PCR and gene editing assays are needed to validate causal regulatory links, a standard analytical pipeline for developmental transcriptomic research of *O. mykiss* [24,25]. Thirdly, standard GO and KEGG databases lack teleost-specific customized functional annotations. In addition, only short-read transcriptome sequencing was adopted in this research, and full-length transcript capture was not performed [49,60]. Subsequent research will integrate tissue-specific transcriptome profiling and CRISPR-mediated gene editing to validate core inflammatory and steroid signaling cascades. Multi-timepoint qRT-PCR assays will be conducted to evaluate the phenotype-associated candidate transcripts efficiency of *slitrk3*, *itpr1* and *grm5*, whose phenotype-associated candidate transcripts potential has been confirmed in prior teleost skeletal malformation studies [42]. Taken together, cross-species multi-omic evidence confirms the conserved biological roles of calcium and neuroactive ligand signaling cascades, while subtype-specific IGF and steroid signaling axes mediate distinct malformation phenotypes. Additional functional assays are needed to elucidate their causal regulatory relationships. Complementary multi-tissue transcriptomic profiling across distinct organs will further uncover tissue-dependent molecular responses in triploid rainbow trout, as demonstrated by recent gill-focused salinity-challenge transcriptome work [61]. Furthermore, multi-factor orthogonal physiological assays are valuable for disentangling how aquaculture-related environmental variables shape larval physiological performance [62]. Further qPCR validation using RNA isolated from vertebral-bone tissues of malformed larvae will be performed in follow-up experiments to verify the expression patterns of these candidate genes. Additional functional assays are required to confirm whether these potential pathways are causally associated with the occurrence of spinal deformity and conjoined-twin malformation.

## 5. Conclusions

The present study was the first to perform combined transcriptomic and PPI topological profiling of normal, spinal-deformed and conjoined twin triploid *O. mykiss* larvae. This work identified shared disrupted neuroimmune and cell adhesion cascades common to both malformation phenotypes, alongside phenotype-specific transcriptional programs for spinal-deformed larvae and conjoined twin larvae. Calcium and MAPK potential cascades display distinct osteogenic transcript alterations in spinal-deformed individuals, while steroid biosynthesis signaling shows characteristic transcriptional changes in conjoined twin larvae. Three filtered PPI sub-networks exhibited distinct topological characteristics, and integrated omics screening uncovered three discriminative candidate transcripts, namely *slitrk3*, *itpr1* and *grm5*, with inverse expression patterns across larval phenotypes. Our findings deliver valuable insights into the divergent molecular pathogeneses underlying two typical larval malformations and supply transcriptomic references for teleost embryonic development research. It is also worth noting that all transcriptomic data were obtained from whole-larval homogenates. The transcriptional signatures detected in conjoined-twin larvae may be confounded by altered organ-tissue proportions, rather than solely reflecting molecular events specific to the conjoined-twin malformation. Further tissue-targeted sequencing and CRISPR-mediated functional assays are required to validate the causal regulatory functions of these candidate hub genes and signaling cascades. These candidate genes and potential pathways derived from transcriptomic data still require independent experimental verification, such as qPCR quantification from vertebral bone samples, before they can be applied for molecular diagnosis of larval malformation in triploid *O. mykiss* hatcheries.

## Data Availability

The raw RNA sequencing data generated in this study have been deposited in NCBI under BioProject accession number PRJNA1477098.

## Supplementary Materials

The following supporting information can be downloaded at https://www.mdpi.com/article/10.3390/ani16213358/s1, Table S1. Summary of sequencing quality metrics for nine larval samples; Table S2. Statistics of sequencing read alignment against the reference genome; Table S3. Statistics Table of Differentially Expressed Genes; Table S4. DEG functional annotation; Table S5. GO enrichment analysis of DEGs from SD vs. N comparison; Table S6. GO enrichment analysis of DEGs from CT vs. N comparison; Table S7. GO enrichment analysis of DEGs from SD vs. CT comparison; Table S8. KEGG enrichment results for SD vs. N; Table S9. KEGG enrichment results for CT vs. N; Table S10. KEGG enrichment results for CT vs. SD; Table S11. Full differential expression statistics of the 13 phenotype-associated candidate transcripts across three pairwise comparisons; Figure S1. Normalized rlog expression profiles of the 13 phenotype-associated candidate transcripts across N, SD, and CT triploid *O. mykiss* larvae.
