# Supplementary material for: Transcriptomic Analysis Reveals Candidate Genes and Potential Pathways Associated with Spinal Deformity and Conjoined Twin Malformation in Oncorhynchus mykiss Larvae
Source: Animals (Basel). 2026 Oct 24;16(21):3358. doi: 10.3390/ani16213358 (PMC13762070; doi:10.3390/ani16213358)

Table S1. Summary of sequencing quality metrics for nine larval samples

Sample	Raw reads	Raw bases	Clean reads	Clean bases	Error rate(%)	Q20(%)	Q30(%)	GC content(%)
N_1	43971816	6639744216	43649888	6536490028	0.0123	99.03	95.32	50.78
N_2	38417370	5801022870	38152474	5698716966	0.0123	99.05	95.33	49.86
N_3	38525456	5817343856	38231786	5713417684	0.0122	99.06	95.48	49.83
SD_1	39547628	5971691828	39270142	5877761014	0.0122	99.10	95.54	49.42
SD_2	41872946	6322814846	41572958	6232672558	0.0123	99.05	95.30	49.74
SD_3	41514332	6268664132	41223524	6166482410	0.0123	99.05	95.32	49.39
CT_1	38975206	5885256106	38697992	5795675822	0.0122	99.05	95.39	49.92
CT_2	39736346	6000188246	39470096	5914836680	0.0122	99.08	95.56	50.01
CT_3	41654714	6289861814	41382616	6196814390	0.0122	99.06	95.45	50.30

Table S2. Statistics of sequencing read alignment against the reference genome

Sample	Total reads	Total mapped	Multiple mapped	Uniquely mapped
N_1	43649888	41244408(94.49%)	4935864(11.31%)	36308544(83.18%)
N_2	38152474	35963250(94.26%)	4038811(10.59%)	31924439(83.68%)
N_3	38231786	36022584(94.22%)	4054337(10.60%)	31968247(83.62%)
SD_1	39270142	37181585(94.68%)	3608527(9.19%)	33573058(85.49%)
SD_2	41572958	39367038(94.69%)	3893800(9.37%)	35473238(85.33%)
SD_3	41223524	38912969(94.40%)	3786058(9.18%)	35126911(85.21%)
CT_1	38697992	36417547(94.11%)	3790463(9.79%)	32627084(84.31%)
CT_2	39470096	37303475(94.51%)	3840111(9.73%)	33463364(84.78%)
CT_3	41382616	39081885(94.44%)	4016620(9.71%)	35065265(84.73%)

Table S3. Statistics Table of Differentially Expressed Genes

Comparison	Total DEGs	Upregulated DEGs	Downregulated DEGs
SD vs N	4292	1643	2649
CT vs N	2699	1365	1334
CT vs SD	3601	1816	1785

Table S5. GO enrichment analysis of DEGs from SD vs N comparison

Number	GO ID	Term Type	Description	Ratio_in_study	Ratio_in_pop	Rich Factor	Pvalue	Padjust	Enrichment	Depth	Pop_count	-log10pvalue	-log10padjust
309	GO:0005615	CC	extracellular space	309/2788	1680/30811	0.183928571	7.01237E-35	8.77948E-32	e	2	1680	34.15413539	31.05653106
83	GO:0050804	BP	modulation of chemical synaptic transmission	83/2788	207/30811	0.400966184	1.96552E-33	6.68965E-30	e	6	207	32.70652267	29.17459692
83	GO:0099177	BP	regulation of trans-synaptic signaling	83/2788	207/30811	0.400966184	1.96552E-33	6.68965E-30	e	5	207	32.70652267	29.17459692
106	GO:0097060	CC	synaptic membrane	106/2788	355/30811	0.298591549	2.77146E-29	1.15662E-26	e	4	355	28.55729171	25.93680863
1232	GO:0016020	CC	membrane	1232/2788	11050/30811	0.111493213	2.45144E-21	7.67302E-19	e	2	11050	20.61057801	18.11503367
168	GO:0043005	CC	neuron projection	168/2788	882/30811	0.19047619	1.15295E-20	2.88697E-18	e	4	882	19.93819133	17.53955701
36	GO:0098878	CC	neurotransmitter receptor complex	36/2788	68/30811	0.529411765	3.06926E-20	5.48959E-18	e	5	68	19.51296673	17.26046044
36	GO:0008328	CC	ionotropic glutamate receptor complex	36/2788	68/30811	0.529411765	3.06926E-20	5.48959E-18	e	6	68	19.51296673	17.26046044
155	GO:0098590	CC	plasma membrane region	155/2788	800/30811	0.19375	5.98342E-20	9.36405E-18	e	3	800	19.22305055	17.02853621
104	GO:0034702	CC	monoatomic ion channel complex	104/2788	479/30811	0.217118998	4.07575E-17	4.63895E-15	e	4	479	16.38979226	14.33358062
29	GO:0008066	MF	glutamate receptor activity	29/2788	50/30811	0.58	4.84854E-18	5.45623E-15	e	4	50	17.31438886	14.26310767
34	GO:0098839	CC	postsynaptic density membrane	34/2788	73/30811	0.465753425	5.97053E-17	5.75008E-15	e	6	73	16.22398716	14.24032618
34	GO:0099634	CC	postsynaptic specialization membrane	34/2788	73/30811	0.465753425	5.97053E-17	5.75008E-15	e	5	73	16.22398716	14.24032618
83	GO:0045202	CC	synapse	83/2788	345/30811	0.24057971	8.00071E-17	7.15492E-15	e	3	345	16.09687124	14.14539495
69	GO:0099537	BP	trans-synaptic signaling	69/2788	251/30811	0.274900398	2.03392E-17	2.50156E-14	e	6	251	16.69166578	13.60178991

Number	GO ID	Term Type	Description	Ratio_in_study	Ratio_in_pop	Rich Factor	Pvalue	Padjust	Enrichment	Depth	Pop_count	-log10pvalue	-log10padjust
70	GO:0099536	BP	synaptic signaling	70/2788	258/30811	0.271317829	2.55952E-17	2.50156E-14	e	5	258	16.59184155	13.60178991
111	GO:0004175	MF	endopeptidase activity	111/2788	541/30811	0.205175601	1.73371E-16	1.46325E-13	e	4	541	15.76102255	12.83468011
106	GO:1902495	CC	transmembrane transporter complex	106/2788	521/30811	0.203454894	2.23026E-15	1.86152E-13	e	3	521	14.65164492	12.73013185
106	GO:1990351	CC	transporter complex	106/2788	526/30811	0.201520913	3.29576E-15	2.57893E-13	e	2	526	14.48204468	12.58856033
90	GO:0007154	BP	cell communication	90/2788	401/30811	0.224438903	4.40805E-16	3.00056E-13	e	2	401	15.35575365	12.5227979

Table S6. GO enrichment analysis of DEGs from CT vs N comparison

Number	GO ID	Term Type	Description	Ratio_in_study	Ratio_in_pop	Rich Factor	Pvalue	Padjust	Enrichment	Depth	Pop_count	-log10pvalue	-log10padjust
185	GO:0005615	CC	extracellular space	185/1704	1680/30811	0.110119048	1.44698E-19	0.00000	e	2	1680	18.83953654	15.74193221
80	GO:0004175	MF	endopeptidase activity	80/1704	541/30811	0.147874307	9.78971E-16	0.00000	e	4	541	15.00923037	11.48082793
21	GO:0016712	MF	oxidoreductase activity, acting on paired donors, with incorporation or reduction of molecular oxygen, reduced flavin or flavoprotein as one donor, and incorporation of one atom of oxygen	21/1704	59/30811	0.355932203	2.3965E-12	0.00000	e	4	59	11.62042199	8.569140809
43	GO:0017171	MF	serine hydrolase activity	43/1704	246/30811	0.174796748	1.8912E-11	0.00000	e	3	246	10.72326207	7.796919627
42	GO:0008236	MF	serine-type peptidase activity	42/1704	241/30811	0.174273859	3.56945E-11	0.00000	e	4	241	10.44739855	7.692487014
40	GO:0004252	MF	serine-type endopeptidase activity	40/1704	222/30811	0.18018018	3.60796E-11	0.00000	e	5	222	10.4427382	7.692487014
97	GO:0008233	MF	peptidase activity	97/1704	875/30811	0.110857143	8.59078E-11	0.00000	e	3	875	10.06596763	7.382663231

Number	GO ID	Term Type	Description	Ratio_in_study	Ratio_in_pop	Rich Factor	Pvalue	Padjust	Enrichment	Depth	Pop_count	-log10pvalue	-log10padjust
32	GO:0004497	MF	monooxygenase activity	32/1704	166/30811	0.192771084	5.49295E-10	0.00000	e	3	166	9.260194078	6.634881627
38	GO:0005506	MF	iron ion binding	38/1704	235/30811	0.161702128	2.70244E-09	0.00000	e	7	235	8.568244614	5.994084686
41	GO:0016705	MF	oxidoreductase activity, acting on paired donors, with incorporation or reduction of molecular oxygen	41/1704	275/30811	0.149090909	7.51082E-09	0.00000	e	3	275	8.12431277	5.595910332
37	GO:0046906	MF	tetrapyrrole binding	37/1704	241/30811	0.153526971	1.81247E-08	0.00001	e	3	241	7.741729198	5.254719445
36	GO:0020037	MF	heme binding	36/1704	234/30811	0.153846154	2.65955E-08	0.00001	e	4	234	7.57519116	5.125969968
7	GO:0050649	MF	testosterone 6-beta-hydroxylase activity	7/1704	9/30811	0.777777778	5.10142E-08	0.00001	e	5	9	7.292309106	4.877850021
63	GO:0031012	CC	extracellular matrix	63/1704	557/30811	0.113105925	9.2811E-08	0.00002	e	3	557	7.032400601	4.712947522
63	GO:0030312	CC	external encapsulating structure	63/1704	557/30811	0.113105925	9.2811E-08	0.00002	e	2	557	7.032400601	4.712947522
13	GO:0008395	MF	steroid hydroxylase activity	13/1704	41/30811	0.317073171	1.77446E-07	0.00004	e	4	41	6.750932854	4.398621675
36	GO:0030246	MF	carbohydrate binding	36/1704	271/30811	0.132841328	1.26197E-06	0.00024	e	2	271	5.89895106	3.625821127
79	GO:0006082	BP	organic acid metabolic process	79/1704	762/30811	0.103674541	8.28507E-08	0.00028	e	4	762	7.081703798	3.549778043
258	GO:0016787	MF	hydrolase activity	258/1704	3535/30811	0.072984441	2.5625E-06	0.00046	e	2	3535	5.591335717	3.34168688
22	GO:0005200	MF	structural constituent of cytoskeleton	22/1704	134/30811	0.164179104	4.38069E-06	0.00070	e	2	134	5.358457033	3.15227389

Table S7. GO enrichment analysis of DEGs from SD vs CT comparison

Number	GO ID	Term Type	Description	Ratio_in_study	Ratio_in_pop	Rich Factor	Pvalue	Padjust	Enrichment	Depth	Pop_count	-log10pvalue	-log10padjust

Number	GO ID	Term Type	Description	Ratio_in_study	Ratio_in_pop	Rich Factor	Pvalue	Padjust	Enrichment	Depth	Pop_count	-log10pvalue	-log10padjust
238	GO:0005615	CC	extracellular space	238/2253	1680/30811	0.141666667	1.63757E-23	2.05023E-20	e	2	1680	22.78580092	19.6881966
54	GO:0004252	MF	serine-type endopeptidase activity	54/2253	222/30811	0.243243243	2.36268E-15	7.97639E-12	e	5	222	14.62659591	11.09819347
55	GO:0008236	MF	serine-type peptidase activity	55/2253	241/30811	0.228215768	2.38828E-14	4.03142E-11	e	4	241	13.62191462	10.39454217
55	GO:0017171	MF	serine hydrolase activity	55/2253	246/30811	0.223577236	5.95283E-14	6.69892E-11	e	3	246	13.22527649	10.1739953
36	GO:0072562	CC	blood microparticle	36/2253	125/30811	0.288	4.72523E-13	2.95799E-10	e	2	125	12.32557725	9.529002922
34	GO:0002252	BP	immune effector process	34/2253	115/30811	0.295652174	9.10921E-13	5.05528E-09	e	2	115	12.04051916	8.296254639
21	GO:0001906	BP	cell killing	21/2253	46/30811	0.456521739	1.48532E-12	5.05528E-09	e	2	46	11.82818039	8.296254639
38	GO:0043177	MF	organic acid binding	38/2253	159/30811	0.238993711	5.44057E-11	4.59184E-08	e	3	159	10.26435578	7.338013336
18	GO:0141061	BP	disruption of cell in another organism	18/2253	38/30811	0.473684211	2.71628E-11	4.62243E-08	e	3	38	10.56602529	7.335129535
18	GO:0031640	BP	killing of cells of another organism	18/2253	38/30811	0.473684211	2.71628E-11	4.62243E-08	e	4	38	10.56602529	7.335129535
36	GO:0004866	MF	endopeptidase inhibitor activity	36/2253	155/30811	0.232258065	4.05845E-10	2.74027E-07	e	5	155	9.391639329	6.562206896
80	GO:0004175	MF	endopeptidase activity	80/2253	541/30811	0.147874307	1.71874E-09	8.28921E-07	e	4	541	8.764791045	6.081486647
14	GO:0034605	BP	cellular response to heat	14/2253	27/30811	0.518518519	9.70089E-10	1.10057E-06	e	5	27	9.013188418	5.958383918
36	GO:0061135	MF	endopeptidase regulator activity	36/2253	166/30811	0.21686747	2.96954E-09	1.25315E-06	e	4	166	8.527310634	5.901998183
36	GO:0030414	MF	peptidase inhibitor activity	36/2253	169/30811	0.213017751	4.92903E-09	1.84893E-06	e	4	169	8.307238766	5.733078837
21	GO:0140662	MF	ATP-dependent protein folding chaperone	21/2253	68/30811	0.308823529	8.35852E-09	2.82183E-06	e	2	68	8.077870864	5.549468426

Number	GO ID	Term Type	Description	Ratio_in_study	Ratio_in_pop	Rich Factor	Pvalue	Padjust	Enrichment	Depth	Pop_count	-log10pvalue	-log10padjust
57	GO:0097060	CC	synaptic membrane	57/2253	355/30811	0.16056338	2.41724E-08	3.02639E-06	e	4	355	7.616679714	5.519075385
82	GO:0006952	BP	defense response	82/2253	574/30811	0.142857143	5.44257E-09	4.3137E-06	e	3	574	8.264196032	5.365150404
44	GO:0046906	MF	tetrapyrrole binding	44/2253	241/30811	0.182572614	1.57484E-08	4.83333E-06	e	3	241	7.802763208	5.315753456
23	GO:0044183	MF	protein folding chaperone	23/2253	85/30811	0.270588235	2.73721E-08	7.70068E-06	e	1	85	7.562691892	5.1134707

Table S8. KEGG enrichment results for SD vs N

Number	Pathway ID	Description	Ratio_in_study	Ratio_in_pop	Rich Factor	Pvalue	Padjust	First Category	Second Category
98	map04514	Cell adhesion molecules	98/3269	492/34430	0.1991869918699187	1.2387614991312266e-12	2.056344088557836e-10	Environmental Information Processing	Signaling molecules and interaction
167	map04080	Neuroactive ligand-receptor interaction	167/3269	1066/34430	0.15666041275797374	6.351710785660601e-11	5.27191995209829 84e-09	Environmental Information Processing	Signaling molecules and interaction
54	map04260	Cardiac muscle contraction	54/3269	264/34430	0.20454545454545456	5.175674536366841e-08	2.863873243456318e-06	Organismal Systems	Circulatory system
86	map04814	Motor proteins	86/3269	534/34430	0.16104868913857678	8.483980334430177e-07	3.520851838788523e-05	Cellular Processes	Cell motility
62	map04145	Phagosome	62/3269	367/34430	0.16893732970027248	5.909052212104897e-06	0.000196180533441 88258	Cellular Processes	Transport and catabolism
74	map04261	Adrenergic signaling in cardiomyocytes	74/3269	481/34430	0.15384615384615385	2.4016382705075345e-05	0.0006644532548404178	Organismal Systems	Circulatory system
118	map04010	MAPK signaling pathway	118/3269	868/34430	0.1359447004608295	4.5698987700754825e-05	0.00108371885118	Environmental	Signal transduction

Number	Pathway ID	Description	Ratio_in_study	Ratio_in_pop	Rich Factor	Pvalue	Padjust	First Category	Second Category
							93287	Information Processing	
43	map04540	Gap junction	43/3269	267/34430	0.16104868913857678	0.0004240798050243821	0.00879965595425593	Cellular Processes	Cellular community - eukaryotes
21	map00590	Arachidonic acid metabolism	21/3269	110/34430	0.19090909090909092	0.0014690855429918945	0.02709646668185049	Metabolism	Lipid metabolism
96	map04020	Calcium signaling pathway	96/3269	759/34430	0.12648221343873517	0.002348337215473923	0.03543854343351555	Environmental Information Processing	Signal transduction
18	map00250	Alanine, aspartate and glutamate metabolism	18/3269	92/34430	0.1956521739130435	0.0023362238748387853	0.03878131632232383	Metabolism	Amino acid metabolism
11	map00790	Folate biosynthesis	11/3269	53/34430	0.20754716981132076	0.009983203746212876	0.1381009851559448	Metabolism	Metabolism of cofactors and vitamins
11	map00603	Glycosphingolipid biosynthesis - globo and isoglobo series	11/3269	55/34430	0.2	0.013159365766759164	0.1560324798058587	Metabolism	Glycan biosynthesis and metabolism
8	map00650	Butanoate metabolism	8/3269	34/34430	0.23529411764705882	0.012550042722243169	0.160254391684028l6	Metabolism	Carbohydrate metabolism
8	map00604	Glycosphingolipid biosynthesis - ganglio series	8/3269	35/34430	0.22857142857142856	0.0149470755946759	0.1654143032477466	Metabolism	Glycan biosynthesis and metabolism
8	map00120	Primary bile acid biosynthesis	8/3269	36/34430	0.2222222222222222	0.017659287450073915	0.18321510729451687	Metabolism	Lipid metabolism
13	map00670	One carbon pool by folate	13/3269	74/34430	0.17567567567567569	0.02126665361678646	0.2076626176697972	Metabolism	Metabolism of cofactors and vitamins
13	map00260	Glycine, serine and	13/3269	77/34430	0.1688311688311688	0.0286906635897	0.250665	Metabolism	Amino acid

Number	Pathway ID	Description	Ratio_in_study	Ratio_in_pop	Rich Factor	Pvalue	Padjust	First Category	Second Category
		threonine metabolism			3	40713	7976787872		metabolism
9	map00591	Linoleic acid metabolism	9/3269	47/34430	0.19148936170212766	0.030769918927791767	0.2553903271006717	Metabolism	Lipid metabolism
7	map00430	Taurine and hypotaurine metabolism	7/3269	32/34430	0.21875	0.027917991753817532	0.2574659239518728	Metabolism	Metabolism of other amino acids

Table S9. KEGG enrichment results for CT vs N

Number	Pathway ID	Description	Ratio_in_study	Ratio_in_pop	Rich Factor	Pvalue	Padjust	First Category	Second Category
17	map00591	Linoleic acid metabolism	17/1896	47/34430	0.3617021276595745	2.0675479812566192e-10	3.328752249823156e-08	Metabolism	Lipid metabolism
46	map04145	Phagosome	46/1896	367/34430	0.12534059945504086	1.8869434605256103e-07	1.51898948572311165e-05	Cellular Processes	Transport and catabolism
20	map00140	Steroid hormone biosynthesis	20/1896	100/34430	0.2	4.5915233625439023e-07	2.46411753789856l3e-05	Metabolism	Lipid metabolism
16	map00670	One carbon pool by folate	16/1896	74/34430	0.21621621621621623	2.1559020069886127e-06	8.677505578129166e-05	Metabolism	Metabolism of cofactors and vitamins
19	map00590	Arachidonic acid metabolism	19/1896	110/34430	0.17272727272727273	8.605507262634813e-06	0.00027709733385684096	Metabolism	Lipid metabolism
53	map04814	Motor proteins	53/1896	534/34430	0.09925093632958802	2.785177911793113e-05	0.0007473560729978184	Cellular Processes	Cell motility
13	map00260	Glycine, serine and threonine metabolism	13/1896	77/34430	0.16883116883116883	0.00027752289338663923	0.006383026547892703	Metabolism	Amino acid metabolism
11	map00410	beta-Alanine metabolism	11/1896	60/34430	0.18333333333333333	0.0003866175853	0.007780	Metabolism	Metabolism of other

Number	Pathway ID	Description	Ratio_in_study	Ratio_in_pop	Rich Factor	Pvalue	Padjust	First Category	Second Category
					2	260549	678904686853		amino acids
14	map00250	Alanine, aspartate and glutamate metabolism	14/1896	92/34430	0.15217391304347827	0.0004971914937041339	0.00889420338737395	Metabolism	Amino acid metabolism
16	map00830	Retinol metabolism	16/1896	120/34430	0.13333333333333333	0.00091598264590836	0.014747320599124597	Metabolism	Metabolism of cofactors and vitamins
10	map00052	Galactose metabolism	10/1896	64/34430	0.15625	0.0024928295619812053	0.02866753996278385	Metabolism	Carbohydrate metabolism
11	map00051	Fructose and mannose metabolism	11/1896	74/34430	0.14864864864864866	0.0023331669779006643	0.02889537564938515	Metabolism	Carbohydrate metabolism
14	map00270	Cysteine and methionine metabolism	14/1896	107/34430	0.1308411214953271	0.002231139098031065	0.02993444956525013	Metabolism	Amino acid metabolism
7	map00650	Butanoate metabolism	7/1896	34/34430	0.20588235294117646	0.002197508112843637	0.03216352783343868	Metabolism	Carbohydrate metabolism
13	map00330	Arginine and proline metabolism	13/1896	108/34430	0.12037037037037036	0.0064529274210289676	0.06926142098571092	Metabolism	Amino acid metabolism
7	map00592	alpha-Linolenic acid metabolism	7/1896	42/34430	0.16666666666666666	0.007515719096908869	0.0756269234126455	Metabolism	Lipid metabolism
11	map02010	ABC transporters	11/1896	89/34430	0.12359550561797752	0.009653087832049195	0.0914204200564659	Environmental Information Processing	Membrane transport
6	map00120	Primary bile acid biosynthesis	6/1896	36/34430	0.16666666666666666	0.01299851243825363	0.1101452896083597	Metabolism	Lipid metabolism
12	map00480	Glutathione metabolism	12/1896	105/34430	0.1142857142857142	0.0128531588139	0.114964	Metabolism	Metabolism of other

Number	Pathway ID	Description	Ratio_in_study	Ratio_in_pop	Rich Factor	Pvalue	Padjust	First Category	Second Category
					8	88906	3649473452		amino acids
6	map00910	Nitrogen metabolism	6/1896	38/34430	0.15789473684210525	0.01678832886864505	0.13514604739259267	Metabolism	Energy metabolism

Table S10. KEGG enrichment results for CT vs SD

Number	Pathway ID	Description	Ratio_in_study	Ratio_in_pop	Rich Factor	Pvalue	Padjust	First Category	Second Category
120	map04080	Neuroactive ligand-receptor interaction	120/2586	1066/34430	0.1125703564727955	5.758622585330337e-06	0.00093865548140 88449	Environmental Information Processing	Signaling molecules and interaction
38	map04260	Cardiac muscle contraction	38/2586	264/34430	0.14393939393939395	8.650560231894197e-05	0.00705020658899377	Organismal Systems	Circulatory system
21	map00983	Drug metabolism - other enzymes	21/2586	128/34430	0.1640625	0.0005607435447791831	0.02285029944975171	Metabolism	Xenobiotics biodegradation and metabolism
46	map04145	Phagosome	46/2586	367/34430	0.12534059945504086	0.00045532890016383243	0.024739536908901558	Cellular Processes	Transport and catabolism
48	map04141	Protein processing in endoplasmic reticulum	48/2586	425/34430	0.11294117647058824	0.0031584301318811725	0.08580401858277185	Genetic Information Processing	Folding, sorting and degradation
11	map00053	Ascorbate and aldarate metabolism	11/2586	56/34430	0.19642857142857142	0.0026700804386817372	0.08704462230102464	Metabolism	Carbohydrate metabolism
12	map00982	Drug metabolism - cytochrome P450	12/2586	70/34430	0.17142857142857143	0.005621671061565917	0.13090462614789208	Metabolism	Xenobiotics biodegradation and metabolism
12	map00980	Metabolism of xenobiotics by cytochrome P450	12/2586	77/34430	0.15584415584415584	0.011982633171479651	0.2170188007723537	Metabolism	Xenobiotics biodegradation and metabolism

Number	Pathway ID	Description	Ratio_in_study	Ratio_in_pop	Rich Factor	Pvalue	Padjust	First Category	Second Category
15	map00480	Glutathione metabolism	15/2586	105/34430	0.14285714285714285	0.011830514454989142	0.24104673202040375	Metabolism	Metabolism of other amino acids
2	map00232	Caffeine metabolism	2/2586	3/34430	0.6666666666666666	0.01607147277348349	0.2619650062077809	Metabolism	Biosynthesis of other secondary metabolites
10	map00040	Pentose and glucuronate interconversions	10/2586	64/34430	0.15625	0.020410541047752643	0.3024471082530619	Metabolism	Carbohydrate metabolism
7	map00340	Histidine metabolism	7/2586	40/34430	0.175	0.027879612612699226	0.3786980713224978	Metabolism	Amino acid metabolism
11	map00380	Tryptophan metabolism	11/2586	79/34430	0.13924050632911392	0.033667445878725825	0.3919852627308793	Metabolism	Amino acid metabolism
14	map00330	Arginine and proline metabolism	14/2586	108/34430	0.12962962962962962	0.03152162955245035	0.3952327397730313	Metabolism	Amino acid metabolism
48	map04514	Cell adhesion molecules	48/2586	492/34430	0.0975609756097561	0.03839949980727632	0.4172745645724027	Environmental Information Processing	Signaling molecules and interaction
14	map00240	Pyrimidine metabolism	14/2586	123/34430	0.11382113821138211	0.07797644832093373	0.5526156989700955	Metabolism	Nucleotide metabolism
2	map00780	Biotin metabolism	2/2586	7/34430	0.2857142857142857	0.09193822734476267	0.5550344835998635	Metabolism	Metabolism of cofactors and vitamins
31	map00230	Purine metabolism	31/2586	315/34430	0.09841269841269841	0.07493852761945903	0.5552263637259918	Metabolism	Nucleotide metabolism
12	map00140	Steroid hormone biosynthesis	12/2586	100/34430	0.12	0.0716154742915263	0.555872490929466	Metabolism	Lipid metabolism

Number	Pathway ID	Description	Ratio_in_study	Ratio_in_pop	Rich Factor	Pvalue	Padjust	First Category	Second Category
20	map01232	Nucleotide metabolism	20/2586	192/34430	0.10416666666666667	0.0860304891351575	0.5609187891612268	Metabolism	Global and overview maps

Figure S1. Normalized rlog expression profiles of the 13 phenotype-associated candidate transcripts across N, SD, and CT triploid *O. mykiss* larvae

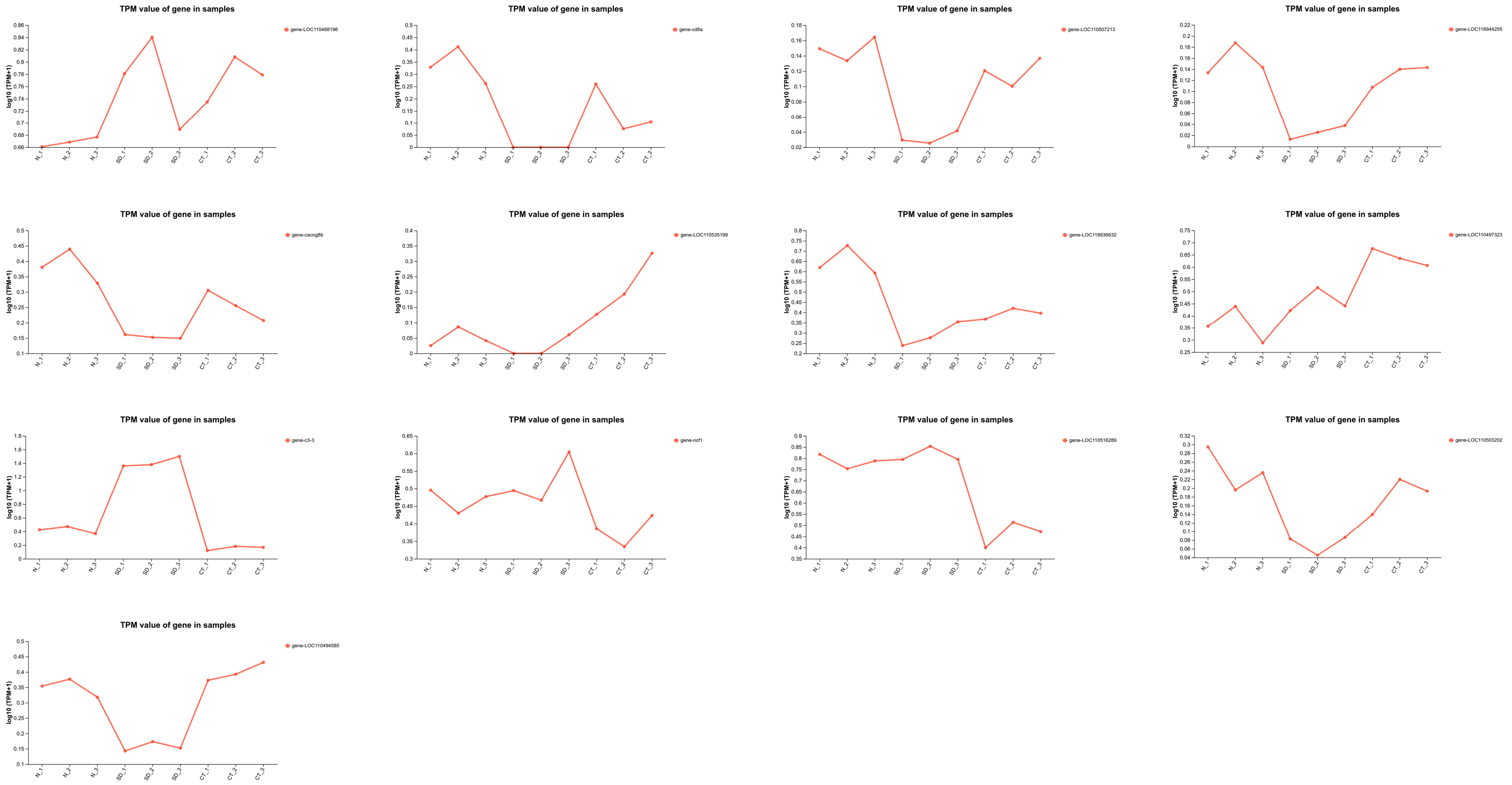

Supplement: Supplementary file 1 [file animals-16-03358-s001.zip › Supplementary Files.pdf]
